# Decreased Tiam1‐mediated Rac1 activation is responsible for impaired directional persistence of chondrocyte migration in microtia

**DOI:** 10.1111/jcmm.18443

**Published:** 2024-06-04

**Authors:** Yi Wu, Wei Liu, Jia Li, Hang Shi, Shize Ma, Di Wang, Bo Pan, Ran Xiao, Haiyue Jiang, Xia Liu

**Affiliations:** ^1^ Research Center of Plastic Surgery Hospital Chinese Academy of Medical Science & Peking Union Medical College Beijing China; ^2^ Department of Auricular Reconstruction, Plastic Surgery Hospital Chinese Academy of Medical Science & Peking Union Medical College Beijing China; ^3^ Key Laboratory of External Tissue and Organ Regeneration Chinese Academy of Medical Sciences Beijing China

**Keywords:** chondrocytes, directional cell migration, microtia, Rac1‐GTP, Tiam1

## Abstract

The human auricle has a complex structure, and microtia is a congenital malformation characterized by decreased size and loss of elaborate structure in the affected ear with a high incidence. Our previous studies suggest that inadequate cell migration is the primary cytological basis for the pathogenesis of microtia, however, the underlying mechanism is unclear. Here, we further demonstrate that microtia chondrocytes show a decreased directional persistence during cell migration. Directional persistence can define a leading edge associated with oriented movement, and any mistakes would affect cell function and tissue morphology. By the screening of motility‐related genes and subsequent confirmations, active Rac1 (Rac1‐GTP) is identified to be critical for the impaired directional persistence of microtia chondrocytes migration. Moreover, Rho guanine nucleotide exchange factors (GEFs) and Rho GTPase‐activating proteins (GAPs) are detected, and overexpression of Tiam1 significantly upregulates the level of Rac1‐GTP and improves directional migration in microtia chondrocytes. Consistently, decreased expression patterns of Tiam1 and active Rac1 are found in microtia mouse models, *Bmp5* ^*se*^ */J* and *Prkra* ^*lear*^ *‐3J/GrsrJ*. Collectively, our results provide new insights into microtia development and therapeutic strategies of tissue engineering for microtia patients.

## 1 INTRODUCTION

Microtia is reported to be one of the most common congenital craniofacial malformations, with a global incidence of 0.83–17.4/10,000.^1^ Microtia is characterized as obvious auricle dysplasia and cartilage tissue loss and is frequently accompanied by hearing loss and other craniofacial developmental defects.^2^ With advanced bioinformatic analysis, recent studies identified several susceptible genes for closely related microtia syndromes to investigate the pathogenesis of certain inherited congenital auricle dysplasias.^3^ These susceptible genes can generally be enriched in specific signalling pathways and biological processes. For instance, the *HOXA2*, *MED12*, *TWIST1*, *GLI3*, *TBX15*, *TFAP2A* and *SIX* genes are involved in embryonic cranial skeleton morphogenesis; *ORC1*, *ORC4*, *ORC6*, *CDC6*, *SMAD4*, *CDK6* and *ATR* are significantly correlated with the cell cycle pathway; *FLNA*, *ITGA7*, *LAMA2* and *COL6* play important roles in ECM‐receptor interaction and focal adhesion formation; and *FGFR2*, *FGFR3* and *ITGA7* are reported to regulate the actin cytoskeleton. Despite these advances, the underlying mechanisms of sporadic nonsyndromic microtia remain poorly understood.

Cranial neural crest cells (NCCs) are a migratory cell population and give rise to the majority of cartilage,^4^ bone,^5^ connective tissue and sensory ganglia^6^ during craniofacial development. According to the most commonly reproduced version, auricle tissue is derived from NCCs that migrate into the first and second branchial arches. Along with embryonic development and concomitant morphological changes, NCCs home towards hillocks and gradually migrate and differentiate into mesenchymal cells, chondrogenic progenitors and chondrocytes until the formation of a recognizable auricle morphology.^7^, ^8^ Therefore, precisely arranged cell migration is crucial for auricles with a complex shape. Previous studies in our laboratory found that chondrocytes derived from microtia migrated deficiently compared with chondrocytes from normal auricles.^9^ Microtia generally characterizes the absence of the hallmark structure of auricles and a decreased size of the ear; therefore, we hypothesized that inadequate cell migration could be the primary cytological basis for the pathogenesis of microtia, and the concrete mechanism needs to be clarified.

Cell migration is a well‐orchestrated dynamic cell biological process that regulates morphogenesis throughout embryonic development, is involved in the majority of physiological and pathological processes and plays an important role in homeostasis maintenance.^10^ In general, it can be simply conceptualized as a cycle process that relies on a highly coordinated spatiotemporal integration of various processes involving intra‐ and extracellular signal transduction, signal pathway activation, cell polarization, cytoskeleton rearrangement‐induced protrusion elongation, focal adhesion formation, extracellular matrix degradation and matrix tunnelling.^11^ For most mesenchymal cells, the initial response to the migration process is to polarize and distinguish the front and back ends of the cell body; afterwards, the cell extends protrusions in the direction of migration to induce oriented movement.^12^, ^13^ Any mistakes would impair the whole program and affect cell function and tissue development. Motility‐related genes/proteins influence different processes of cell motion and orchestrate cell behaviour by mutual regulation.

Rac1 (Ras‐related C3 botulinum toxin substrate 1) is a ubiquitously expressed Rho GTPase family member, and generally be taken as a positive regulator in cell polarization, directional migration and cell proliferation.^14^ Rac1 acts as a molecular switch in cell migration, and integrates multiple signals to orchestrate the cell cytoskeleton dynamics.^15^ The conformational state of Rac1 alternates between the active GTP‐binding state (Rac1‐GTP) and the inactive GDP‐binding state (Rac1‐GDP) with the help of Rho guanine nucleotide exchange factors (Rho GEFs) and Rho GTPase‐activating proteins (Rho GAPs), respectively.^16^ Specifically, Rho GEFs activate Rac1 by stimulating the exchange of a bound GDP nucleotide for GTP, and Rho GAPs catalyse the hydrolysis of the bound GTP returning the Rac1 to an inactive state.^15^ Besides, the phosphorylation of Rac1 would also affect its GTPase activity.^17^ Therefore, the role and activation of Rac1 are strongly influenced by its conformational state, and affect different aspects of directional cell migration.

In the current study, we compared distinct migration parameters of chondrocytes derived from microtia and normal auricular cartilage and found that microtia chondrocytes showed impaired directional persistence during cell migration. A cell motility PCR array was used to identify significantly downregulated candidate motility‐related genes in microtia chondrocytes, including *RAC1*, *ENAH*, *VASP*, *MMP14* and *PXN*. Moreover, we presented evidence that activation of Rac1 was deficient in microtia chondrocytes, and Rho GEF Tiam1 was responsible for Rac1 activation to induce impaired persistence of directional migration. Finally, we verified the expression pattern of Tima1 and active Rac1 in microtia mouse models.

## 2 MATERIALS AND METHODS

### 2.1 Human auricular cartilage samples

Human auricular cartilage samples were obtained from microtia patients following the approved guidelines set by the ethical committee at Plastic Surgery Hospital (Institute), Chinese Academy of Medical Sciences and Peking Union Medical College. Written informed consent was obtained from each patient to harvest and utilize clinical samples for research purposes. Human auricular cartilage tissues were harvested from auricular reconstruction surgery and divided into a normal auricle group (Nor, *n* = 20) and a microtia group (mic, *n* = 120).

### 2.2 Isolation and cultivation of human auricular chondrocytes

Auricular cartilage was cut into small pieces (<1 mm^3^) after detaching skin and connective tissue and then digested in trypsin containing 0.25% EDTA (HyClone, USA) for 30 min at 37°C with shaking at 80–100 × rpm prior to changing 0.2% collagenase IV (Sigma, USA) for 8–12 h. Cells were harvested after filtering through a 70‐μm cell strainer (BD Falcon, Germany), and the suspensions were centrifuged at 300 × *g* for 5 min. Chondrocytes were collected and cultured with high‐glucose Dulbecco's modified Eagle's medium (DMEM, Gibco, USA) supplemented with 10% FBS (Gibco, USA) and 1× penicillin–streptomycin solution (HyClone, USA). Unattached cells were washed off with PBS after culturing for 72 h, and the medium was changed every 3 days. Chondrocyte morphology was evaluated under microscopy.

### 2.3 Histochemical and immunofluorescence

Auricular cartilage was fixed with 4% paraformaldehyde, dehydrated, embedded in paraffin and cut into thin slices (4 μm). Tissue sections were dewaxed and hydrated before staining with haematoxylin and eosin. After sealing with neutral resin, images were captured by a Leica DM3000 microscope (Leica Microsystems Gmbh, Wetzlar, Germany).

Chondrocytes were cultured on confocal dishes (NEST Biotechnology Co., Ltd. China), were fixed and permeabilized, followed by blocking with 5% normal goat serum (Nanjing Jiancheng Bioengineering Institute, China) at room temperature. Cells were incubated with the following primary antibodies overnight at 4°C: Rac1 Mouse mAb (ab33186), Ena/VASP‐like Rabbit polyAb (ab204835), MMP14 Rabbit mAb (ab51074), PXN Rabbit mAb (ab32084), Rac1 + Cdc42(phospho S71) Rabbit polyAb (ab203884) (Abcam, Cambridge, UK) and active Rac1‐GTP Mouse mAb (Neweastbio, USA). TRITC phalloidin (CA1610, Solarbio, China) and AlexaFluor‐coupled antibodies were utilized to visualize actin and for secondary detection, including goat anti‐mouse IgG (H + L) Alexa Fluor 488 (ab150117), goat anti‐rabbit IgG (H + L) Alexa Fluor 488 (ab150077), goat anti‐mouse IgG (H + L) Alexa Fluor 594 (ab150116), goat anti‐rabbit IgG (H + L) Alexa Fluor 594 (ab150080), goat anti‐mouse IgG (H + L) Alexa Fluor 647 (ab150115) and goat anti‐rabbit IgG (H + L) Alexa Fluor 647 (ab150079) (Abcam, Cambridge, UK). Cells were mounted with antifade mounting medium (S2100, Solarbio, China) after localizing to the nucleus with DAPI (C1005, Beyotime Biotechnology, China). Confocal images were acquired and quantified by an FV1200 confocal microscope with a 60 × 1.3 NA oil objective lens (Olympus, Japan).

### 2.4 Transmission electron microscope (TEM) imaging of auricle cartilage

Auricular cartilage was cut into small pieces (<1 mm^3^) and fixed with a mixture of 2.5% glutaraldehyde and 2% paraformaldehyde (Sigma, USA) before changing reduced osmium treatment (rOTO). Gradient ethanol and acetone solutions were used for dehydration. After resin embedding, ultrathin sections were cut in an ultramicrotome (Leica‐EM UC7, Germany). Lead citrate (0.1%) and uranyl acetate (10%) were used to enhance contrast. Subcellular structures were imaged by transmission electron microscopy (Hitachi H‐7650, Japan).

### 2.5 Wound healing assay

Chondrocytes cultured on 6‐well plates were carefully scratched with a 200‐μL sterile plastic pipette tip after 100% confluence and washed with PBS. DMEM without FBS was used to culture cells for 72 h. The wound areas were imaged with a Leica DM3000 microscope and quantified with ImageJ (National Institutes of Health, USA).

### 2.6 Transwell assay

Primary chondrocytes were treated with trypsin containing 0.25% EDTA (HyClone, USA), resuspended in serum‐free DMEM, and then seeded at 1.5 × 10^4^ cells/well in 24‐well Transwell® cell culture inserts (8 μm, BD Falcon™, Germany). DMEM with 10% FBS was added to the lower compartment. After culturing for 12 and 24 h, cells remaining on the upper sides of the semipermeable membrane were removed with cotton swabs. Cells on the lower sides were fixed with 4% paraformaldehyde and stained with crystal violet. The migrated cells were imaged under a microscope in five randomly chosen fields, and then crystal violet was eluted by acetic acid and measured at 590 nm on an EnSpire™ Multimode Plate Reader (PerkinElmer, USA). Three independent experiments were conducted, and the results were calculated in different fields in every test.

### 2.7 Cell migration high‐content analysis

Chondrocytes were seeded at 3 × 10^3^ cells/well in a 96‐well cell carrier ultramicroplate (PerkinElmer, USA). After attachment, cell movement was captured in 3 × 3 fields per well by a high‐content analysis system (Operetta CLS™, PE, USA) with a 10 × 0.3 NA air objective lens for 48 h. Every chondrocyte was acquired and tracked under a bright field channel, digital phase contrast (DPC) channel and GFP channel. Cell migration was quantified by Harmony 4.9 software (PE, USA) with a cell‐tracking protocol.

In the tracking protocol, five important indicators represent different aspects of cell migration, including displacement, accumulated distance, average speed, straightness and degrees. Displacement of cell migration represents the exact range from the start position towards the end position during the observation window, which means the effective movement of cells to a specific location. The accumulated distance of cell migration is the sum of recorded every step during the observation window. The average speed is the mean value of the speed recorded in every time phase. Notably, the accumulated distance and average speed could indicate cell motor ability and cell activity, but contain no directional indication, which means that the cells might move around quickly but without approaching the destination. Straightness is the ratio of the migration displacement to accumulated distance, and reflects the duration in the fixed direction. Rotation degrees refer to the angle at which the cell migration trajectory deviates from a fixed direction. In the current study, the dispersion of straightness and rotation degrees could reflect the directional persistence of cell migration.

### 2.8 Oris™ cell migration assay and cell IQ analysis

Centripetal migration was monitored with an Oris™ Cell Migration Assay (Platypus Technologies, USA). According to the manufacturer's protocol, chondrocytes were seeded in a 96‐well Oris™ microplate. The Cell Seeding Stoppers were removed to reveal a 2‐mm diameter unseeded region in the centre of each well after cells 100% converged. Centripetal migration was monitored with serum‐free medium for 48 h by a Cell‐IQ Live Cell Real‐time Imaging Analysis System (MLF, Chip‐Man, Finland), and cell trajectories and motility parameters were analysed by an accompanying analysis system.

### 2.9 Cell motility PCR array and real‐time polymerase chain reaction (PCR)

Total RNA was extracted with TRIzol reagent (Invitrogen, USA), trichloromethane and isopropanol and reverse transcribed with oligo dT (Promega, USA), M‐MLV reverse transcriptase (Promega, USA) and dNTPs (Sigma–Aldrich, Germany) according to the manufacturer's instructions. Real‐time PCR was performed with a Light Cycler® 480 SYBR Green Master and Light Cycler® 480 system (Roche, Switzerland) according to the manufacturer's instructions. Primers were designed by Primer Premier 5 software (Canada) and are presented in Table S1. The cell motility PCR array (Table S2) was a 96‐well microplate precoated with corresponding gene probes (Shanghai Xingyuan Biotechnology Co. Ltd, China) and detected by a Light Cycler® 480 system. Pre‐denaturation was performed for 10 min, amplification was performed for 40 cycles (95°C, 10 s), and annealing and extension were performed (60°C, 30 s). The relative expression of each gene was normalized by comparison with the reference *GAPDH* and measured with the 2^−△△CT^ method.

### 2.10 Western blot

Cellular protein was harvested in RIPA lysis buffer containing 1 mmol/L phenylmethylsulfonyl fluoride (PMSF, Beyotime Biotechnology, China). BCA Protein Quantification Kits (Sigma, USA) were used to determine protein concentrations. Equivalent amounts of protein were separated by electrophoresis on a 10% SDS–PAGE gel and then transferred to an Immobilon‐NC membrane (0.45 μm HATF00010, Millipore, USA). After blocking with 5% fat‐free milk, the membranes were incubated with primary antibodies, including Rac1 mouse mAb, ENAH rabbit mAb (ab124685), Ena/vasp‐like rabbit polyAb, MMP14 rabbit mAb, PXN rabbit mAb, Rac1 + Cdc42 (phospho S71) rabbit polyAb, Tiam1 rabbit polyAb (ab211518) and anti‐tubulin loading control mouse mAb (ab56676) (Abcam, Cambridge, UK). Affinity‐purified goat anti‐mouse IgG + IgM H&L (HRP) (ab47827) and goat anti‐rabbit IgG H&L (HRP) (ab97080) (Abcam, Cambridge, UK) were utilized for secondary detection. The SuperSignal® West Pico Trial Kit (Thermo Fisher Scientific) was applied for protein detection.

### 2.11 Plasmid and virus transfection

The DNA sequences of *RAC1* (NM_006908.4), *TIAM1* (NM_001353689.1) and Raichu‐Rac1 were synthesized and integrated into the pCDH‐CMV‐MCS‐EF1‐copGFP lentiviral vector (pCDH‐GFP, System Biosciences, Palo Alto, CA, USA). On the basis of the Rac1‐pCDH plasmid, the glutamine at site 61 was mutated to leucine to obtain a constitutively active Rac1‐Q61L‐pCDH plasmid. According to Itoh RE, the FRET plasmid Raichu‐Rac1 consists of YFP (amino acids [aa] 1–239), a spacer (Leu‐Asp), CRIB of PAK1 (aa 68–150), a spacer (Ser‐Gly‐Gly‐Thr‐Gly‐Gly‐Gly‐Gly‐Thr), Rac1 (aa 1–176), a spacer (Gly‐Gly‐Arg), CFP (aa 1–237), a spacer (Gly‐Arg‐Ser‐Arg) and the CAAX box of Ki‐Ras (aa 169–188). The acceptor and the donor needed to be modified as YFP (Thr66Gly, Val69Leu, Ser73Ala, Aln70Lys and Thr204Tyr) and CFP (Lys27Arg, Tyr67Trp, Asp130Gly, Asn147Iso, Met154Thr, Val164Ala, Asn165His and Ser176Gly), respectively.^18^

An EndoFree® Plasmid Maxi Kit (12362, QIAGEN, French) was used to amplify plasmids. According to the manufacturer's protocol, lentiviral vectors were packaged with psPAX2 and pMD2. G (Addgene, Cambridge, MA) in 293T cells using jetPRIME® Transfection Regent (PolyPlus‐Transfection, French). The supernatants containing virus were collected and concentrated before infecting chondrocytes with 5 μg/mL polybrene (Millipore, USA).

### 2.12 G‐LISA Rac1 activation assay

Cellular protein was harvested rapidly (<10 min) on ice, according to the manufacturer's instructions, which is essential for accurate and reproducible results. Equivalent amounts of protein (0.5 mg/mL) were loaded to examine the content of GTP‐bound Rac1 in each group with the G‐LISA® Rac1 Activation Assay Biochem Kit™ (BK128, Cytoskeleton, USA). The results were collected at 490 nm on a PerkinElmer EnSpireTM Multimode Plate Reader, designated lysis buffer‐only wells as the assay blank and the results were corrected with the positive control by the standard protein attached to the kit.

### 2.13 Fluorescence resonance energy transfer (FRET)

Chondrocytes were transfected with the Raichu‐Rac1 biosensor. After 72 h of transfection, chondrocytes were imaged with a FLUOVIEW FV1200 confocal fluorescence microscope (IX8 Olympus, Japan). A UPLSAPO 60× NA 1.3 oil immersion objective was used to collect the fluorescence signal. For FRET Acceptor Photobleach, using CFP/YFP/FRET, the following filter sets were necessary: CFP:475‐500, SDM5105, YFP:515‐615. Chondrocytes were photobleached with a 515‐nm laser for 3 s. Before and after exposure, images of YFP/CFP ratios were captured and calculated to represent FRET efficiency. All images and videos were acquired and analysed after subtracting background and correcting alignment channels with FV1200 Software (FV10‐ASW4.1).

### 2.14 Statistical analysis

Statistical significance was determined using a two‐tailed Student's *t*‐test and one‐way ANOVA multiple comparisons using GraphPad Prism 8.4.0 software (GraphPad Software, Inc., San Diego, CA). The level of significance was set at *p* < 0.05. Data are shown as the mean ± SEM.

## 3 RESULTS

### 3.1 Microtia chondrocytes showed impaired directional persistence during cell migration

**FIGURE 1:**
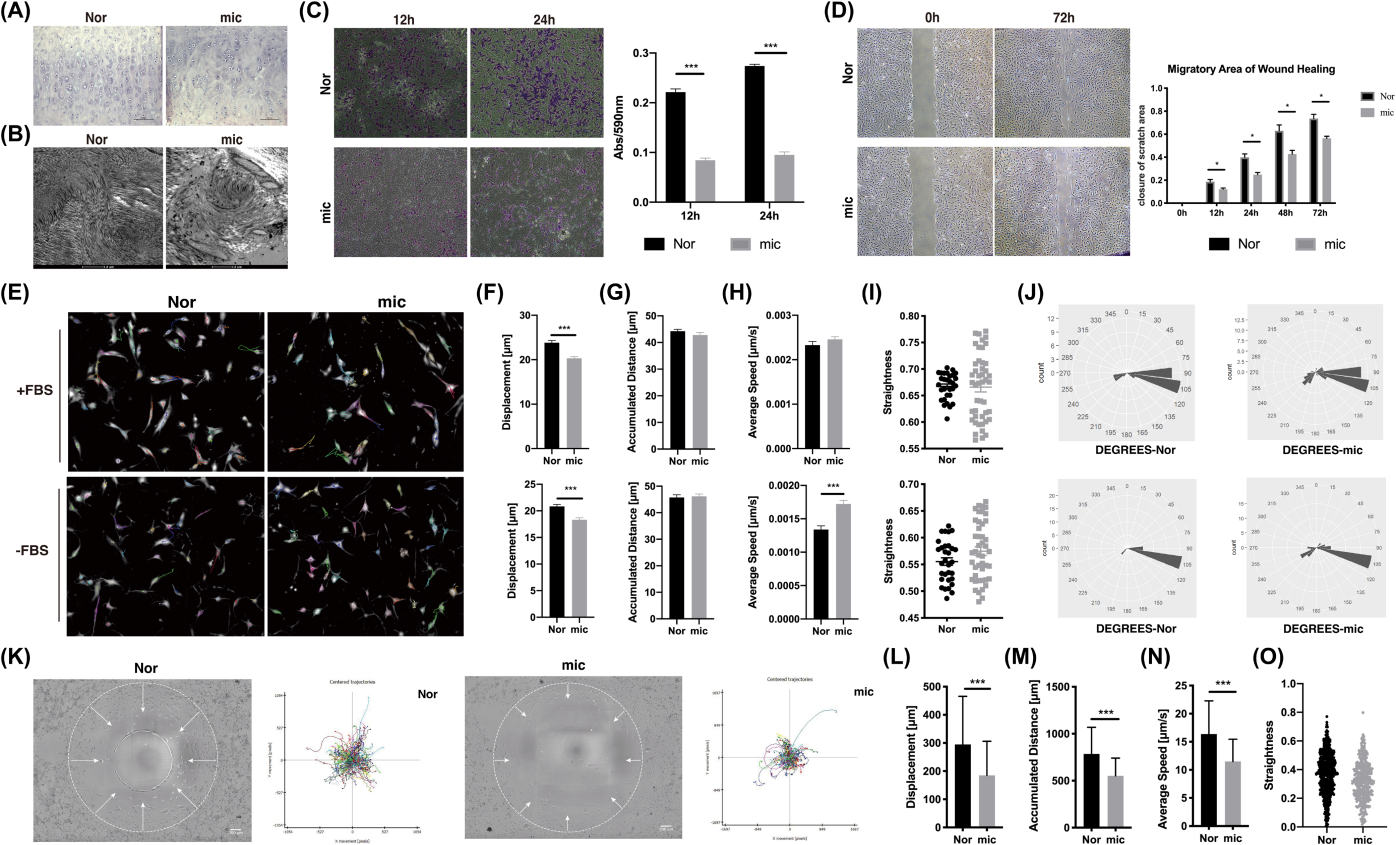
Microtia chondrocytes showed impaired directional persistence during cell migration. (A) Representative haematoxylin and eosin (H&E) staining of normal auricle and microtia cartilage. Scale bars, 100 μm. (B) Representative photographs of transmission electron microscopy of normal auricle and microtia cartilage. Scale bars, 2 μm. (C) The Transwell assay and statistical analysis of Nor (*n* = 3) and mic (*n* = 9). (D) The wound healing assay and migratory area statistics of Nor (*n* = 3) and mic (*n* = 3). (E–J) Trajectories and analysis of spontaneous cell migration with the CLS high‐content cell imaging system, and the coefficient of variation of straightness and rotation degrees were shown as rose plots. Nor (*n* = 10), mic (*n* = 16). (K–O) The Oris™ Cell Migration Assay and statistical analysis of Nor (*n* = 4) and mic (*n* = 4), data were analysed using single cell tracking. The white circle indicates the initial state of cell migration, and the white arrow represents the cell migration stream. Scale bars, 200 μm. Nor indicates normal chondrocytes, mic indicates microtia chondrocytes. Data were analysed using unpaired two‐tailed Student's *t* test. Values are presented as the mean ± SEM. *Indicates *p* < 0.05 and ***indicates *p* < 0.001.

In the current study, our results showed that chondrocytes were arranged in a disorderly manner in microtia cartilage, whereas in normal auricular cartilage, they exhibited a typical columnar arrangement (Figure 1A). Transmission electron microscopy further revealed neater collagen fibres in normal auricular cartilage; in contrast, irregularly crimped collagen fibres were observed in microtia cartilage (Figure 1B). In addition, both the Transwell assay and wound healing assay proved that the migration capability of microtia chondrocytes (mic) was significantly deficient compared to that of normal chondrocytes (Nor) (Figure 1C,D).

To clarify the dynamic characteristics of spontaneous cell motility, we captured and analysed the trajectories of chondrocytes with a high‐content imaging system at a low‐cell density (Figure 1E, Movie S1). In our study, the accumulated distance of chondrocytes showed no significant difference between the microtia and normal groups (Figure 1G). The average speed of microtia chondrocytes was higher than that of normal chondrocytes in medium without FBS (Figure 1H). These two parameters were used to assess cell motor ability. However, cell displacement representing the exact range from the start position towards the end point was decreased in microtia chondrocytes compared to normal chondrocytes (Figure 1F). The dispersion of straightness and rotation degrees in cell migration were higher in microtia chondrocytes (Figure 1I,J). Straightness and rotation degrees refer to the duration in the fixed direction and the migration turning angles, respectively, which indicate directional persistence during cell migration. Similar trends of spontaneous chondrocyte motility were observed under both FBS abundant and starvation conditions. Therefore, our results first demonstrated impaired directional persistence of microtia chondrocyte migration.

Moreover, directional migration of cells was assessed with the Oris™ Cell Migration Assay and Cell IQ Imaging System (Figure 1K, Movie S2). When cells migrated centripetally, microtia chondrocytes showed significantly defective performances in all aspects, including displacement, accumulated distance, average speed and straightness (Figure 1L–O).

### 3.2 The expression of motility‐related genes decreased with aberrant localization in microtia chondrocytes

**FIGURE 2:**
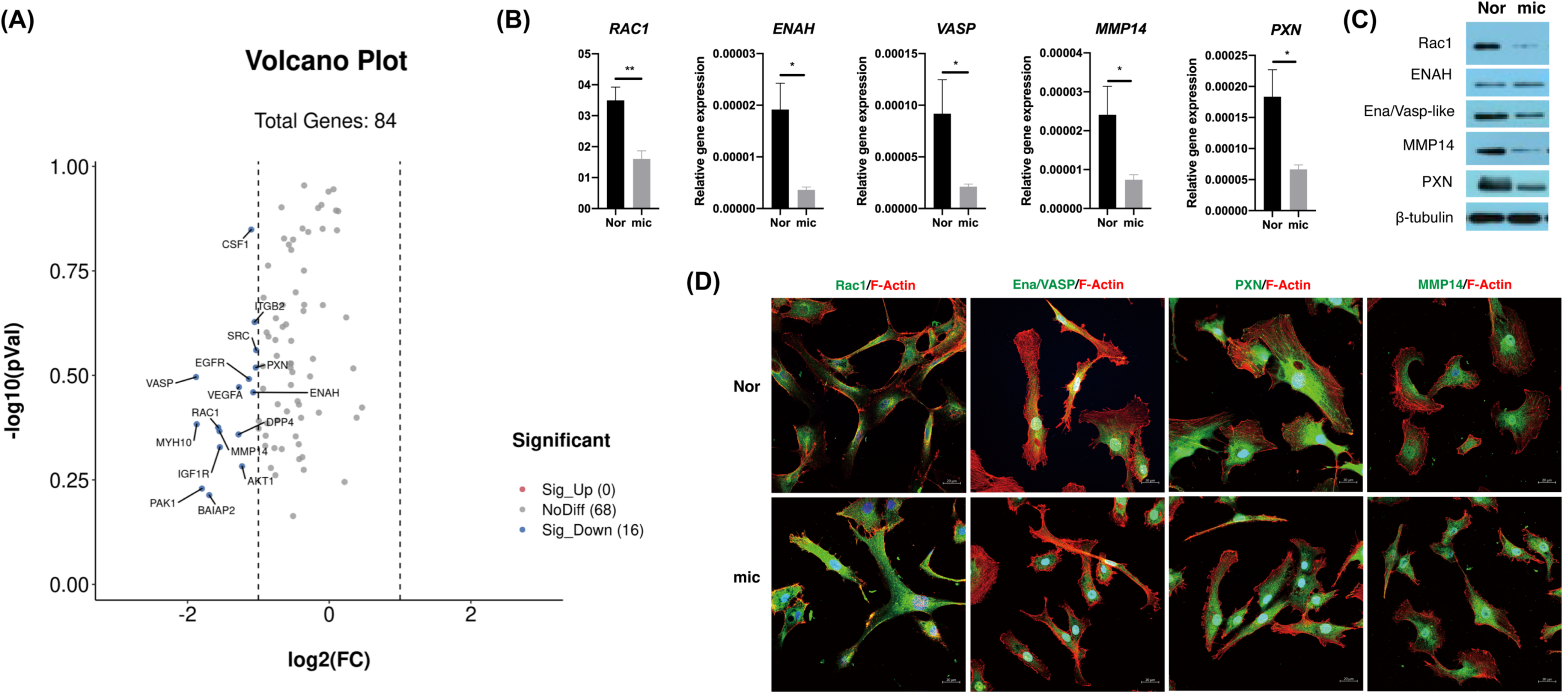
The expression of motility‐related genes decreased with aberrant localization in microtia chondrocytes. (A) Volcano plot of differentially expressed motility‐related genes in Nor (*n* = 3) and mic (*n* = 3) (Log_2_(FC) < −1). (B) The mRNA expression of *RAC1*, *ENAH*, *VASP*, *MMP14* and *PXN* between Nor (*n* = 13) and mic (*n* = 12). Relative gene expression was normalized to *GAPDH*. (C) The protein level of Rac1, ENAH, Ena/VASP‐like, MMP14 and PXN in normal and microtia chondrocytes. (D) Representative immunofluorescence staining of five candidate proteins in normal and microtia chondrocytes. Experiment was repeated three times independently. Scale bars, 20 μm. Nor indicates normal chondrocytes, mic indicates microtia chondrocytes. Data were analysed using two‐tailed Student's *t* test. Values are presented as the mean ± SEM. *Indicates *p* < 0.05 and **indicates *p* < 0.01.

Using a cell motility PCR array, we found that the expression of 16 motility‐related genes was significantly decreased in microtia chondrocytes (Log_2_(FC) < −1), while the expression of no motility‐related gene was upregulated (Log_2_(FC) > 1) (Figure 2A, Table S2). Five differentially expressed genes, RAC1, ENAH, VASP, PXN and MMP14, were confirmed by real‐time PCR and western blot (Figure 2B,C). In addition, their specific distributions were observed in normal and microtia chondrocytes by immunofluorescence staining (Figure 2D). Specifically, Rac1 and Ena/VASP aggregated on protrusions of the cell leading edge, and MMP14 and PXN were mainly localized at the rear of the cells. Their localizations in normal chondrocytes correspond with individual functions. Rac1 induces cell polarization and orientation during migration,^10^ ENAH and VASP can associate with actin filaments to protect them from being capped by capping protein (CP),^19^, ^20^ PXN is well known as a scaffolding molecule within focal adhesion (FA) complexes,^21^ and MMP14 generally helps to degrade extracellular matrix to form movement pathways.^22^ In contrast, in microtia chondrocytes, Rac1 and PXN were diffused in the cytoplasm, and Ena/VASP and MMP14 were concentrated in the perinuclear area. Therefore, these unorchestrated motility‐related genes might be responsible for the impaired directional persistence of microtia chondrocytes. Based on the above, the microtia chondrocytes showed impaired directional persistence during cell migration, which responds to the role of Rac1. So we chose Rac1 as the candidate gene for further exploration.

### 3.3 Rac1 showed abnormal conformational states in microtia chondrocytes

**FIGURE 3:**
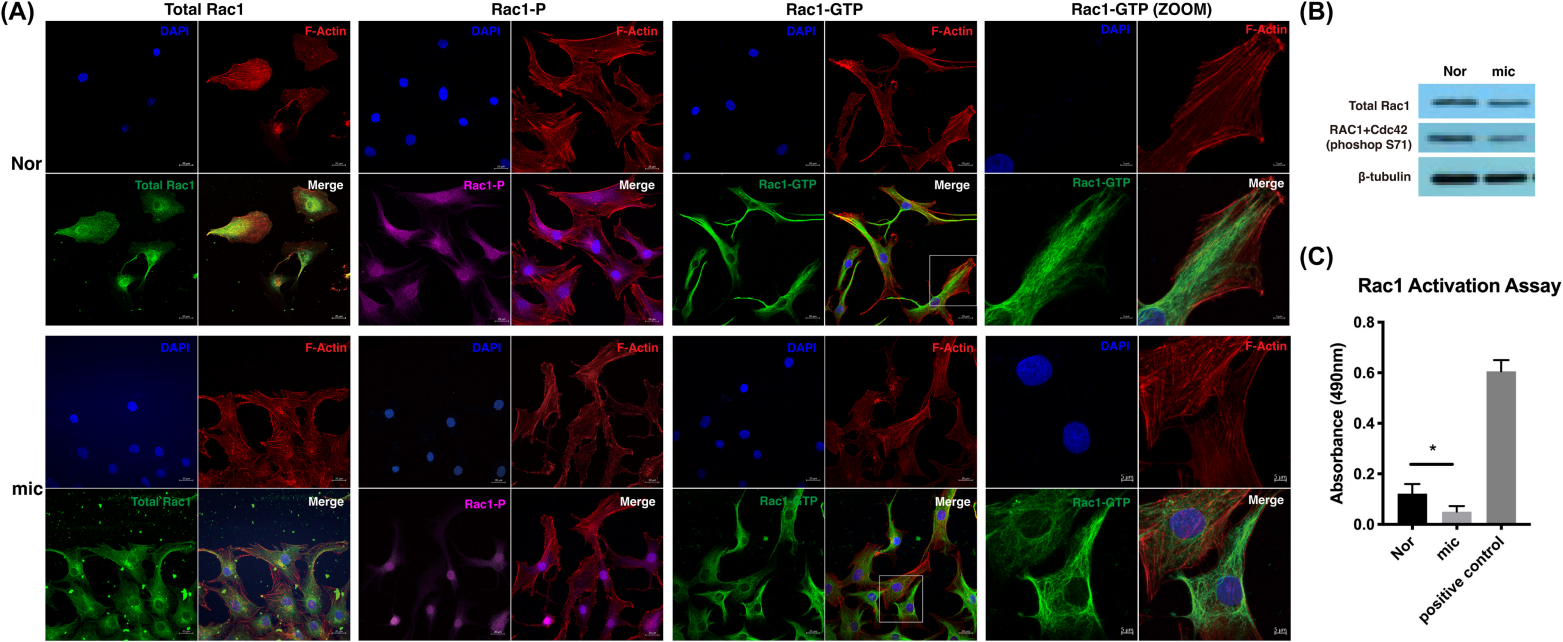
Rac1 showed abnormal conformational states in microtia chondrocytes. (A) Representative immunofluorescence imaging of different states of Rac1 in migratory chondrocytes based on wound healing assay. Scale bars, 20 μm, 5 μm. (B) The protein expression level of total Rac1 and phosphorylated Rac1 (S71) in microtia chondrocytes and normal chondrocytes. (C) The active Rac1 level of normal chondrocytes (*n* = 3) and microtia chondrocytes (*n* = 3). The relative content of active Rac1 was normalized by comparison with total protein, and the results were corrected with the positive control by the standard protein attached to the kit (*n* = 3). Nor indicates normal chondrocytes, mic indicates microtia chondrocytes. Data were analysed using two‐tailed Student's *t* test. Values are presented as the mean ± SEM. *Indicates *p* < 0.05.

It has been reported that Rac1 interacts with ENAH, VASP, PXN and MMP14, and different conformational states of Rac1 are located in diverse places in the cell to adapt for their specific functions. We then detected the distribution and content of total Rac1, phosphorylated Rac1 and active Rac1 in microtia and normal chondrocytes. When chondrocytes migrated to the scratching area, total Rac1 aggregated in the leading edge of pseudopodia in normal chondrocytes but diffused in the cytoplasm in microtia chondrocytes (Figure 3A). Phosphorylated Rac1 (Rac1‐P) expressed in both cytoplasm and nucleus of normal chondrocytes, whereas Rac1‐P majorly accumulated in nucleus of microtia chondrocytes. In addition, in normal chondrocytes, active Rac1 (Rac1‐GTP) concentrated at the front of protrusions to induce cell polarization and actin monomer recruitment. However, in microtia chondrocytes, the collection of active Rac1 and the formation of actin fibres were out of sync at the protrusions of the leading edge. Moreover, the expression of phosphorylated Rac1 in microtia chondrocytes was deficient compared with that in normal chondrocytes (Figure 3B). G‐LISA analysis showed that the expression level of active Rac1 (Rac1‐GTP) in microtia chondrocytes was 2.4 times lower than that in normal chondrocytes (Figure 3C).

### 3.4 Active Rac1 was critical for directional persistence of microtia chondrocyte migration

**FIGURE 4:**
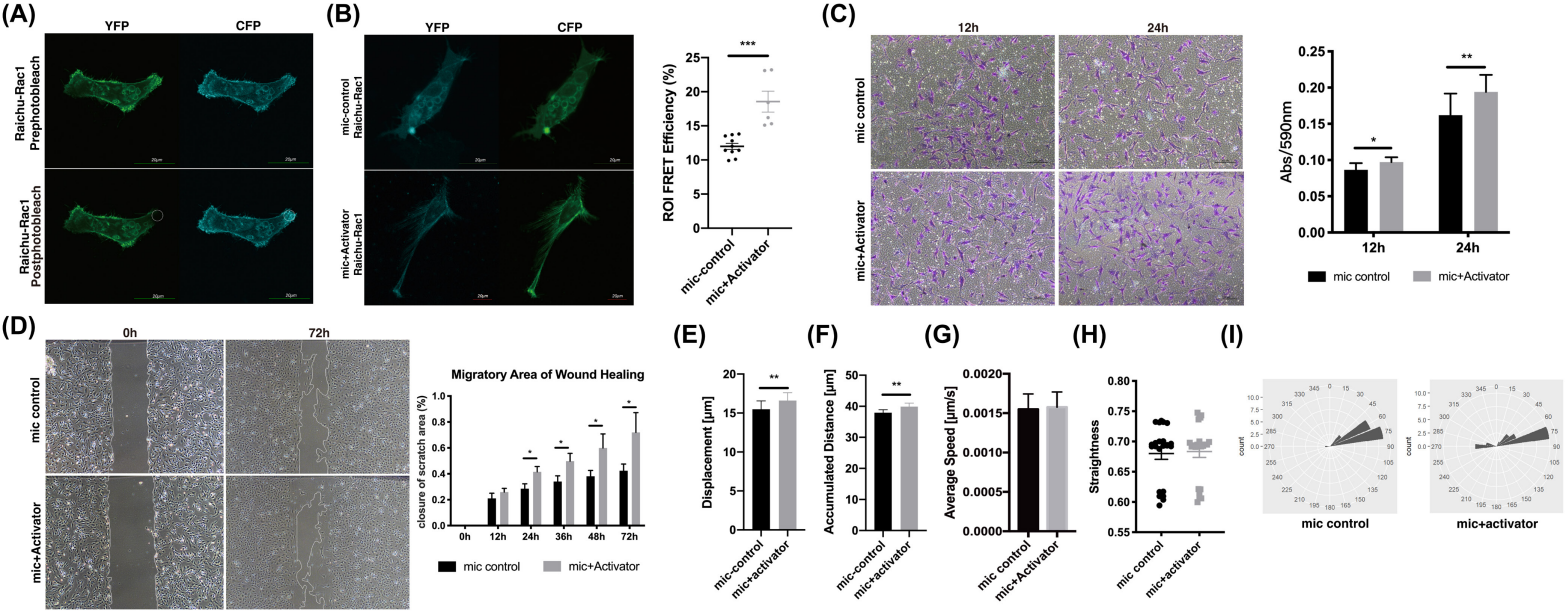
Active Rac1 was critical for directional persistence of microtia chondrocyte migration. (A) Spatiotemporal maps of active Rac1 dynamics were imaged with a FRET‐based Rac1 biosensor in microtia chondrocytes. The white circle indicates photobleached area (ROI). Scale bars, 20 μm. (B) The FRET efficiency of microtia chondrocytes after 5 min of stimulation. Scale bars, 20 μm. (C) Transwell assay and statistical analysis of microtia chondrocytes (*n* = 4) with stimulation. (D) The wound healing assay and migratory area statistics of microtia chondrocytes (*n* = 3) and the corresponding stimulation group. (E–I) Trajectories and analysis of spontaneous cell migration of microtia chondrocytes and the corresponding stimulation group (*n* = 4). Mic indicates microtia chondrocytes. Data were analysed using two‐tailed Student's *t* test. Values are presented as the mean ± SEM. *Indicates *p* < 0.05, **indicates *p* < 0.01, and ***indicates *p* < 0.001.

Active Rac1 was reported to be responsible for cell polarization and related to directional persistence of cell migration. To explore how active Rac1 influences the migration of microtia chondrocytes, we exogenously activated Rac1 in microtia chondrocytes. We also constructed a FRET‐based Rac1 biosensor to produce spatiotemporal maps of active Rac1 dynamics in microtia chondrocytes^18^ (Figure 4A). The Raichu‐Rac1 probe labelled Rac1 binding with GTP, and the peak activity of signals aggregated at the cell membrane margin, which is consistent with a report in MEFs.^13^ After Rac1 activator II treatment, the Raichu‐Rac1 probe showed massive filopodia formation in microtia chondrocytes after approximately 5 min of stimulation (Figure 4B). The FRET efficiency was approximately 10%–14% in control microtia chondrocytes and then significantly increased to 15%–23% after activator treatment. Enhanced active Rac1 aggregation, massive filopodia, rapid extension of protrusions and significant movement of microtia chondrocytes after stimulation can be observed in the supplementary video (Movie S3). Both Transwell and wound healing assays demonstrated that the migration ability of microtia chondrocytes was significantly increased after activator treatment (Figure 4C,D). The displacement and accumulated distance of microtia chondrocytes were distinctly increased after activator stimulation (Figure 4E,F, Movie S4), even though there was no significant change in the average speed and straightness (Figure 4G,H). All the results above indicated that insufficient activation of Rac1 contributed to defective migration of microtia chondrocytes, which could be improved by a Rac1 activator.

### 3.5 Rho GEF Tiam1 activated Rac1 and improved directional migration in microtia chondrocytes

**FIGURE 5:**
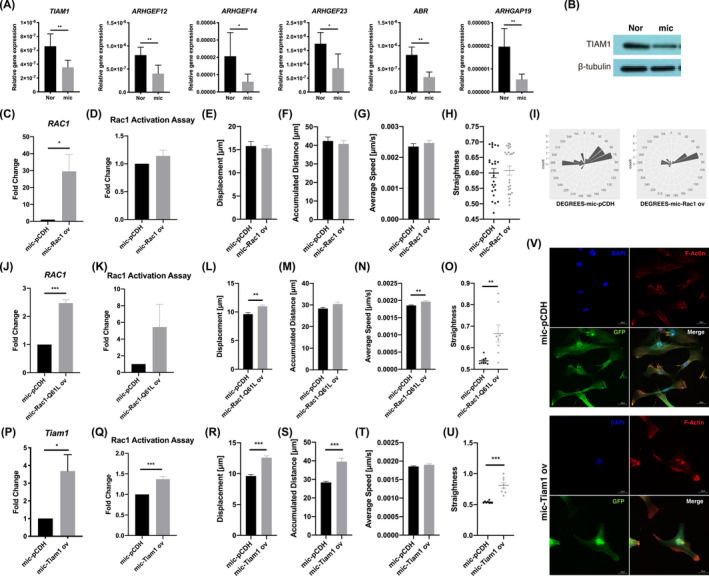
The Rho guanine nucleotide exchange factor Tiam1 activated Rac1 and improved directional migration in microtia chondrocytes. (A) The mRNA expression of *TIAM1*, *ARHGEF12*, *ARHGEF14*, *ARHGEF23*, *ABR* and *ARHGAP19* in mic (*n* = 9) and Nor (*n* = 4). (B) The protein level of TIAM1 in mic and Nor. (C) The mRNA expression of *RAC1* in microtia chondrocytes after overexpression (*n* = 9). (D) The active Rac1 level of microtia chondrocytes (*n* = 3) after Rac1 overexpression. (E–I) Trajectories and analysis of spontaneous cell migration with the CLS high‐content cell imaging system in microtia chondrocytes after Rac1 overexpression. (J) The mRNA expression of *RAC1* in microtia chondrocytes after Rac1‐Q61L overexpression (*n* = 4). (K) The active Rac1 level in microtia chondrocytes after Rac1‐Q61L overexpression (*n* = 4). (L–O) Trajectories and analysis of spontaneous cell migration in microtia chondrocytes after Rac1‐Q61L overexpression (*n* = 3). (P) The mRNA expression of *Tiam1* in microtia chondrocytes after Tiam1 overexpression (*n* = 4). (Q) The active Rac1 level in microtia chondrocytes after Tiam1 overexpression (*n* = 3). (R–U) Trajectories and analysis of spontaneous cell migration in microtia chondrocytes after Tiam1 overexpression (*n* = 8). (V) Representative immunofluorescence imaging of microtia chondrocytes after Tiam1 overexpression. Scale bars, 20 μm. Nor indicates normal chondrocytes, mic indicates microtia chondrocytes. Data were analysed using two‐tailed Student's *t* test. Values are presented as the mean ± SEM. *Indicates *p* < 0.05, **indicates *p* < 0.01, and ***indicates *p* < 0.001.

To investigate the mechanism of insufficient activation of Rac1 in microtia chondrocytes, we screened the relative expression of Rho GEFs and Rho GAPs in normal auricular and microtia chondrocytes (Figure 5A). The results showed that the expression of four GEFs, *TIAM1*, *ARHGEF12*, *ARHGEF14* and *ARHGEF23*, and two GAPs, *ABR* and *ARHGAP19*, was significantly lower in microtia chondrocytes than in normal chondrocytes. As Tiam1 is a well‐known Rac1‐specific GEF, its expression at the protein level was verified, and a decreased level was confirmed in microtia chondrocytes (Figure 5B).

To rescue the directional migration of microtia chondrocytes, we further overexpressed Rac1, Tiam1 and Rac1‐Q61L (a constitutively active Rac1 mutant^23^, ^24^), and named as mic‐Rac1, mic‐Tiam1 and mic‐Rac1‐Q61L, respectively (Figure 5C,J,P). After Rac1 overexpression, the active Rac1 level was not upregulated in microtia chondrocytes (Figure 5D), and the displacement, accumulated distance, average speed and straightness were not significantly changed (Figure 5E–H), though the dispersion of rotation degrees decreased from 0.71587 to 0.67270 (Figure 5I, Movie S5). After Rac1‐Q61L overexpression, the active Rac1 level increased approximately 5.44 times but no statistical significance (Figure 5K), the displacement, average speed and straightness of microtia chondrocyte migration were significantly improved (Figure 5L–O). Notably, the active Rac1 level significantly increased after overexpressing Tiam1 (Figure 5Q), and the displacement, accumulated distance and straightness were distinctly improved (Figure 5R–U). Additionally, the stretching actin filaments aggregated at the end of protrusions to form significant actin bundles in Tiam1 overexpressed microtia chondrocytes (Figure 5V). These results further confirmed that the Tiam1‐mediated Rac1‐GTP/GDP cycle was impaired in microtia chondrocytes.

### 3.6 Microtia mouse models exhibited abnormal expression patterns of Tiam1 and active Rac1 in ear cartilage tissues

**FIGURE 6:**
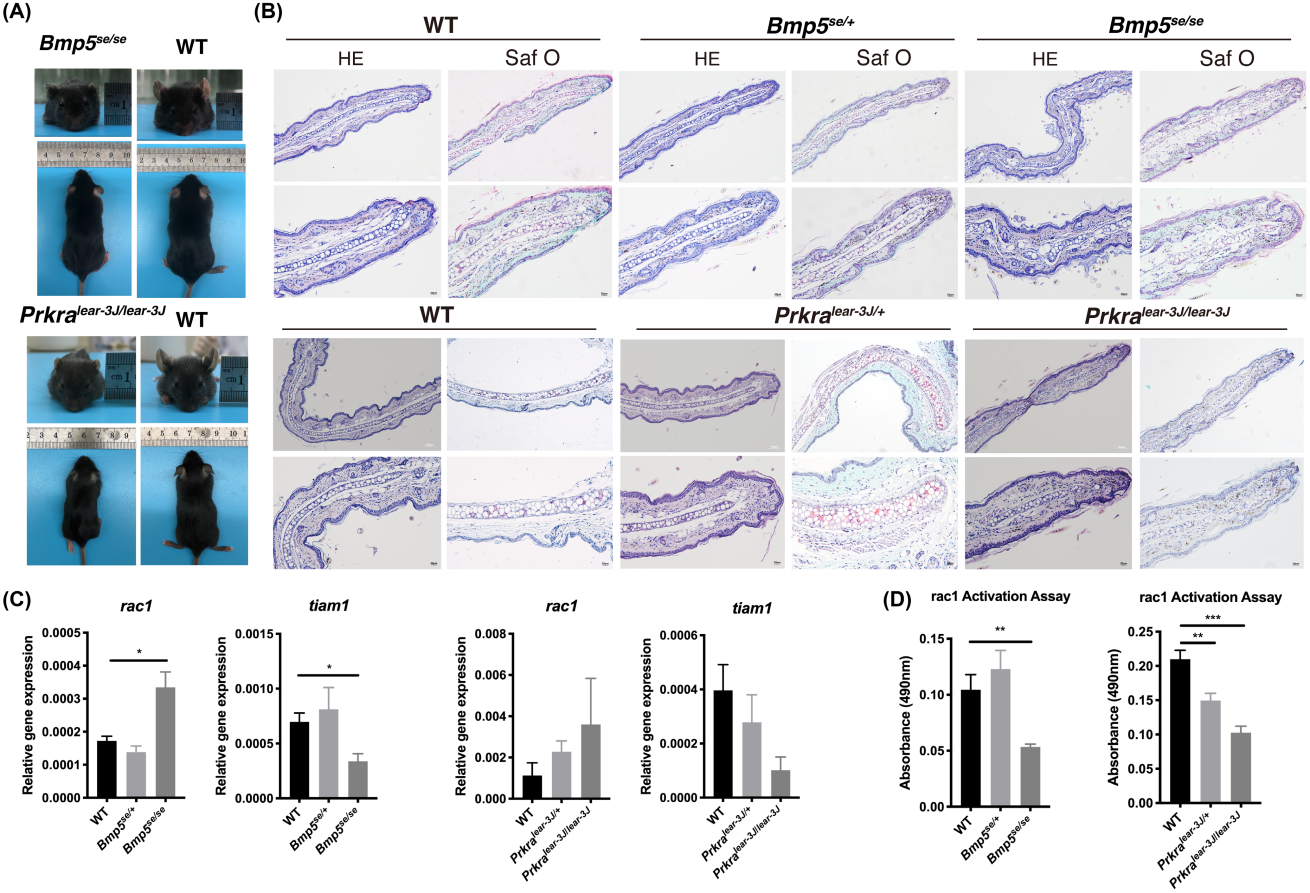
Microtia mouse models exhibited an abnormal expression pattern of Tiam1/Rac1 in ear tissues. (A) The gross view of homozygous mutants of the *Bmp5* ^*se*^ */J* and *Prkra* ^*lear*^ *‐3J/GrsrJ* microtia mouse models and wild‐type control. (B) Representative haematoxylin and eosin (H&E) staining and Saf O staining of auricular cartilage in *Bmp5* ^*se*^ */* ^*se*^ and *Prkra* ^*lear‐3J/lear‐3J*^ individuals, corresponding heterozygotes and WT mouse. Scale bars, 100 μm, 20 μm. (C) The *rac1* and *tiam1* mRNA expression of *Bmp5* ^*se/se*^ (*n* = 4) mutant, *prkra* ^*lear‐3J/lear‐3J*^ mutant (*n* = 4), corresponding heterozygotes and WT individuals (*n* = 4), respectively. (D) The active rac1 level of *Bmp5* ^*se/se*^ individuals (*n* = 6), *prkra* ^*lear‐3J/lear‐3J*^ individuals (*n* = 6), corresponding heterozygotes (*n* = 6) and WT control (*n* = 9). Data were analysed using one‐way ANOVA for multiple comparisons. Values are presented as the mean ± SEM. *Indicates *p* < 0.05, **indicates *p* < 0.01, and ***indicates *p* < 0.001.

To detect whether the abnormal expression pattern of Tiam1/Rac1 could be recapitulated in microtia mouse models, we assessed *Bmp5* ^*se*^ */J* and *Prkra* ^*lear*^ *‐3J/GrsrJ* mice from JAX lab.^25^, ^26^ Both homozygous mutants *Bmp5* ^*se/se*^ and *Prkra* ^*lear‐3J/lear‐3J*^ exhibited obvious small ears and dwarfs, and the cartilage layers of the mutant ear showed fractured and distorted construction; in contrast, the auricular cartilage of the heterozygous mutant and WT was more orderly and homogeneous (Figure 6A,B).

*Bmp5* ^*se/se*^ ear cartilage presented significantly higher expression of *rac1* and lower expression of *tiam1* than the WT control and heterozygous mutant cartilage (Figure 6C). *Prkra* ^*lear‐3J/lear‐3J*^ individuals showed similar trends in the mRNA expression of *rac1* and *tiam1* as *Bmp5* ^*se/se*^ individuals, although without significant differences compared to the WT control and heterozygous mutant. However, the active rac1 level in the ear cartilage of both homozygous mutants was distinctly lower than that of the WT control and heterozygous mutant (Figure 6D). These results suggested that the impaired rac1 activation induced by tiam1 contributed to failed ear cartilage development in microtia mouse models.

## 4 DISCUSSION

The main characteristics of microtia are the decreased size and the loss of elaborate structure in the affected ear. Our previous study reported that microtia chondrocytes exhibited a significantly decreased capability of cell migration accompanied by irregularly crimped collagen fibres. In the current study, we demonstrated that the deficient migration of microtia chondrocytes manifested as decreased directional persistence during migration. To investigate potential motility‐related genes involved in impaired microtia chondrocyte migration, we screened 84 genes and confirmed five candidate genes with significantly lower expression, including *RAC1*, *ENAH*, *VASP*, *MMP14* and *PXN*. It has been reported that the Ras signalling pathway contributes to microtia^27^ and that Rac1 positively regulates polarization and orientation during the migration process ascribed to its Rho GTPase activity. Therefore, we investigated the conformational states of Rac1 in microtia chondrocytes and found that the level of active Rac1 was significantly defective. The screening of GEFs and GAPs showed that the expression of Tiam1, the well‐known Rac1‐specific Rho GEF, was decreased in microtia chondrocytes. The active Rac1 level was upregulated after Tiam1 overexpression in microtia chondrocytes, and directional persistence of cell migration was significantly improved. Furthermore, the microtia mouse models *Bmp5* ^*se*^ */J* and *Prkra* ^*lear*^ *‐3J/GrsrJ* were analysed, and abnormal expression profiling of Tiam1 and active Rac1 was found in the defective ear cartilage of homozygous mutants. Collectively, our results provide new insight into microtia pathogenesis and the development of abnormal shapes.

Cell migration is an underlying principle for various biological processes and governs distinct physiological and pathological courses.^10^ Persistently directional migration is generally taken as a part of development or regenerative programs and occurs in collective cell streams.^28^, ^29^ During embryogenesis, appropriate cell migration and arrangement lay the foundation for the normal architecture, which crucially depends on the directional persistence of stem cells.^30^, ^31^ The migration of mesenchymal cells has been studied in‐depth and characterized by a polarity axis from the intrinsic front to the rear and persistent actin polymerization at the leading edge of protrusions.^32^, ^33^ Mesenchymal cells move slowly to be optimized for fidelity, allowing modifications and corrections through the surrounding environment, which aims to correspond with developmental morphogenesis.^34^ Microtia is a typical dysformation of auricular cartilage with the loss of precise ear architecture and disordered arrangement of chondrocytes, which we believe results from inappropriate migration of ear cartilage development‐related NCC cells and differentiated mesenchymal cells, chondrogenic progenitors and chondrocytes. Here, we first reported impaired persistent directional migration in microtia chondrocytes and decreased expression of motility‐related genes, including *RAC1*, *ENAH*, *VASP*, *PXN* and *MMP14*.

The migratory behaviour of mesenchymal cells in vitro was concluded to be in two phases.^11^ First, cells undergo a diffusive phase extending protrusions in different directions but cannot sustain elongation in a persistent direction and move forwards. In the second phase, cells break the symmetry stage and become polarized, which gives cells the capability to move along a specific directional trajectory and populate new territories.^35^, ^36^ Well‐orchestrated directional cell migration is based on the establishment of a front to rear polarity axis,^37^ and cell polarity is modulated by complex signalling cascades that centre around Rho small GTPase activation.^38^ Symmetric breaking is mediated through two oscillating systems based on Rac activity: PIP3 levels initiate Rac1 activation, and actin polymerization maintains Rac1 activation.^39^, ^40^ Moreover, directional cell migration involves more sophisticated signal interactions and polarization persistence than spontaneous cell motility. In 2014, Krause M proposed that there are three major mechanisms at play in controlling lamellipodial persistence and their influence on directional persistence.^20^ The first is the balance between actin filament branching and elongation induced by WAVE‐mediated ARP2/3 activity and ENA/VASP‐mediated ARP2/3 activity, respectively. The second is a positive‐feedback loop that promotes GEFs and GTPase recruitment and actin polymerization, involving both a convergent elongation model and a de novo nucleation model.^19^ The last is Rac1‐mediated negative feedback loops, ECM confinement and steering. In conclusion, to adjust the efficiency and stability of actin rearrangement, Rac1 regulates interactions of downstream effectors causing lamellipodial oscillations, and only the most persistent protrusions define a leading edge associated with effective migration.

Rac1 is ubiquitously expressed in organisms, is involved in the Rho small GTPase family and is well established to positively regulate cell polarization, directional migration and proliferation, as it has been reported to be a member of the noncanonical planar cell polarity Wnt pathway.^41^, ^42^ Rac1 performs different states in the cell, total Rac1, phosphorylated Rac1 (Rac1‐P) and active Rac1 (Rac1‐GTP). Total Rac1 ubiquitously distributes in the cell and plays as a substrate for distinct modifications. Rac1‐P contributes to cell proliferation by entering the nucleus to stimulate the transcription factors TCF/LEF (T‐cell factor/lymphoid enhancement factor).^16^ According to previous reports, Rac1 actually acts as the ‘molecular switch’ in the process of cell migration through a cycle between its inactive (Rac1‐GDP) and active (Rac1‐GTP) conformations.^13^, ^15^ To initiate and maintain directional cell migration, elevated Rac1 at the leading edge of the cell is activated to provide the major force for directional migration.^14^, ^43^ Our results demonstrated that the decreased activation of Rac1 was crucial for the impaired dynamic properties of directional migration in microtia chondrocytes, which could not be rescued by overexpression of Rac1, similar to the Rac1 knockout results in Mahalakshmi Ramadass's reports.^44^ Rac1 is activated through interaction with diffuse B‐cell lymphoma (Dbl) family GEFs (putatively β‐Pix, Dock3, DOCK1 (180), Asef, Vav2, Tiam1) and inactivated through interaction with the GAP family.^45^ Rac1‐GTP accumulates in the membrane of the leading edge to activate the Rac1‐SCAR/WAVE‐ARP2/3‐ENA/VASP signal cascade and mediate the reconstruction of the actin skeleton, which is consistent with our results.^46^ Orchestrated spatiotemporal turning of the GDP/GTP cycle of Rac1 is essential for persistent directional cell migration.

Tiam1 (T‐lymphoma invasion and metastasis 1) is a specific Rho GEF for Rac1. Mature Rac1 interacts with PIP2/PIP3 at the plasma membrane with the help of Rho GEFs, adhesion molecules, RTKs and scaffold proteins, which are critical for Rac1 localization and activation at the leading edge of the migrating membrane.^16^, ^47^ Individual GEFs and GAPs localize separately depending on which proteins they form complexes with, highlighting the significance of these distinct signalling units in defining functional outcomes.^45^ Unlike Vav2, which ubiquitously stimulates global GTP loading and activation of Rac1, Tiam1 mainly facilitates the recruitment of Rac1 to the membrane and subsequent activation.^48^ Tiam1‐mediated Rac1 signalling is required for establishing and maintaining cell polarity. Tiam1 interacts with the Par (partitioning defective) polarity complex through Par3 and commonly leads to the formation of a gradient of active Rac1 at the leading edge of protrusions to establish stable cell polarity and induce persistent directional cell migration.^49^ Our study further confirmed that in microtia chondrocytes, the stable activation of Rac1 at the cell leading edge and directional persistence could be improved through Tiam1 rescue. We also confirmed the disordered cartilage arrangement and abnormal expression pattern of Tiam1 and active Rac1 in *Bmp5* ^*se*^ */J* and *Prkra* ^*lear*^ *‐3J/GrsrJ* homozygous mutations,^50^ indicating similar cytological pathogenesis during the dysmorphogenesis of microtia mouse models.

In addition, Rac1 has been regarded as the centre of the regulatory network in the process of cell migration. ENAH (also known as Mena, mammalian Protein Enabled Homolog) and VASP (vasodilator‐stimulated phosphoprotein) belong to the ENA/VASP family and reduce the ratio of branches per unit length of actin filament by antagonizing the function of the actin‐related protein 2/3 (ARP2/3) complex during protrusion extension.^19^ Notably, ARP2/3‐induced dynamic actin reorganization is activated by the WAVE regulatory complex (WRC) downstream of Rac subfamily GTPase signalling,^51^ which gives rise to interference between Rac family proteins and the ENA/VASP family. PXN (paxillin) belongs to the paxillin gene family of adapter proteins and is well known as a scaffolding molecule within focal adhesion (FA) complexes.^21^ Paxillin senses the physical properties of the microenvironment, translates them into biochemical signals and then recruits other molecules to adhesions, in turn leading to Rac1 activation^52^, ^53^ and organizing actin rearrangement.^54^ MMP14 (matrix metalloproteinase 14, also known as MT1‐MMP) plays an important role in cell migration, lineage predetermination and specific morphogenesis by regulating ECM remodelling, and defects or disorders in its expression and/or localization result in failed EMT,^55^ developmental malformation or even death.^56^ Previous studies report that the LOX‐1‐MT1‐MMP axis is crucial for RhoA and Rac1 activation.^57^ We hypothesized that deficient expression of *ENAH*, *VASP*, *PXN* and *MMP14* contributes to decreased Rac1 activation and impaired dynamic proprieties in microtia chondrocytes, which could be further investigated in the future.

Prevalent whole‐genome sequencing and whole‐exome sequencing based on microtia families showed that mutated genes and SNP sites were concentrated in many signalling pathways, including the Ras signalling pathway, regulation of actin cytoskeleton and focal adhesion. Recently, researchers have obtained iPSC‐NCCs by inducing fibroblasts from patients with CHARGE syndrome in vitro. Sequencing revealed that CHD7 (an identified mutated gene of CHARGE syndrome) regulated the migration of NCCs via *FOXD1*, affecting the development of organs.^58^ Moreover, a study in melanoma demonstrated that the downstream factor of *FOXD1* regulating cell movement is *RAC1B*, which contains an additional exon 3b compared to *RAC1*, renders it constitutively active.^59^ Altogether, these data indicate that syndromic microtia, family inheritance and sporadic cases share the same cytological behaviour of decreased directional persistence of cell migration due to impaired expression patterns of Tiam1 and active Rac1.

## 5 CONCLUSION

In summary, we first demonstrate that the directional persistence of chondrocytes migration is decreased in microtia, and further find a decreased Tiam1‐mediated aberrant expression and localization of active Rac1 in microtia chondrocytes. Additionally, similar expression patterns of Tiam1 and active Rac1 are observed in microtia mouse models. This study may lead to a better understanding of microtia pathogenesis, and provide a foundation for future investigations into potential prenatal diagnosis and therapeutic strategies.

## AUTHOR CONTRIBUTIONS

**Yi Wu:** Conceptualization (equal); data curation (lead); formal analysis (equal); investigation (equal); writing – original draft (equal). **Wei Liu:** Data curation (equal); resources (equal). **Jia Li:** Data curation (equal); investigation (equal). **Hang Shi:** Data curation (equal); investigation (equal). **Shize Ma:** Data curation (equal); investigation (equal). **Di Wang:** Investigation (equal); resources (equal). **Bo Pan:** Conceptualization (equal); resources (equal). **Ran Xiao:** Conceptualization (equal); funding acquisition (equal); project administration (equal); supervision (equal); validation (equal); writing – review and editing (equal). **Haiyue Jiang:** Funding acquisition (equal); project administration (equal); resources (equal); supervision (equal). **Xia Liu:** Conceptualization (equal); funding acquisition (lead); investigation (equal); project administration (equal); resources (equal); supervision (equal); validation (equal); writing – review and editing (equal).

## CONFLICT OF INTEREST STATEMENT

The authors declare no conflicts of interest.

## Supporting information


Table S1

Table S2

Movie S1

Movie S2

Movie S3

Movie S4

Movie S5

Data S1

## Data Availability

All data generated or analysed during this study are included in this published article. Additional data related to this are available from the corresponding authors upon reasonable request.
