# Supplementary material for: Decreased Tiam1‐mediated Rac1 activation is responsible for impaired directional persistence of chondrocyte migration in microtia
Source: J Cell Mol Med. 2024 Jun 4;28(11):e18443. doi: 10.1111/jcmm.18443 (PMC11149491; doi:10.1111/jcmm.18443)
Supplement: Supplementary file 1 — Table S1 [file JCMM-28-e18443-s007.pdf]

Supplementary Table 1. Primers Sequences

Gene	Primer 5'-3'
ABR	F: GTCCTGGATCGACACCCTCT
	R: TCAGCTTGGCACACAGTAGG
ARHGAP19	F: AGGCACAGAGTGAAGGGGAG
	R: CAATCTCGCACACTCCCGAA
ARHGEF12	F: GACCCCACAGATAGCTCCTC
	R: TGTCCAATGACTGACGGCTC
ENAH	F: GACACTTGTCTCCCGTCTCC
	R: GCAGCTCTTGCCTGACAGAT
GAPDH	F: CTCTGCTCCTCCTGTTCGAC
	R: TTAAAAGCAGCCCTGGTGAC
ARHGEF14 / MCF2L	F: GAGGTTTTGGCTGAGGACTGA
	R: GTGTGCTTGGAAACCGCATT
MMP14	F: GTGGTCTCGGACCATGTCTC
	R: GGTAGCCATATTGCTGTAGCC
PXN	F: AAAGTTGCGGGGCATAGAC
	R: GTAGACTCCAAGTCCGCCAG
RAC1	F: CTGATGCAGGCCATCAAGT
	R: TCTCCAGGAAATGCATTGGT
TIAM1	F: GCTGGGCAAGTGGAAAAAGG
	R: GGGAAGGCTCTCGGTTTTGA
ARHGEF23 / TRIO	F: GGAAGTCACGGGAAGGACTC
	R: GCACTCCAACACTCCACGTA
VASP	F: GAACCCCTGAACCTCCAGC
	R: ATAAAGCATCACAGTGGCCC
rac1 (Mus)	F: CGCAGACAGTTGGAGACACAT
	R: GTGGTGTCGCACTTCAGGAT
tiam1 (Mus)	F: GAGCTGCCAAACCCCAAAAG
	R: CGCCTTCTCACTCCGATCTC
β-actin (Mus)	F: GATCAAGATCATTGCTCCTCCTG
	R: AGGGTGTAAAACGCAGCTCA
