# Supplementary material for: Decreased Tiam1‐mediated Rac1 activation is responsible for impaired directional persistence of chondrocyte migration in microtia
Source: J Cell Mol Med. 2024 Jun 4;28(11):e18443. doi: 10.1111/jcmm.18443 (PMC11149491; doi:10.1111/jcmm.18443)
Supplement: Supplementary file 2 — Table S2 [file JCMM-28-e18443-s006.pdf]

	1	2	3	4	5	6	7	8	9	10	11	12
A	ACTN1	ACTN3	ACTN4	ACTR2	ACTR3	AKT1	ARF6	ARHGDIA	ARHGEF7	BAIAP2	BCAR1	CAPN1
B	CAPN2	CAV1	CDC42	CFL1	CRK	CSF1	CTTN	DIAPH1	DPP4	EGF	EGFR	ENAH
C	EZR	FAP	FGF2	HGF	IGF1	IGF1R	ILK	ITGA4	ITGB1	ITGB2	ITGB3	LIMK1
D	MAPK1	MET	MMP14	MMP2	MMP9	MSN	MYH10	MYH9	MYL9	MYLK	PAK1	PAK4
E	PFN1	PIK3CA	PLAUR	PLCG1	PLD1	PRKCA	PTEN	PTK2	PTK2B	PTPN1	PXN	RAC1
F	RAC2	RASA1	RDX	RHO	RHOA	RHOB	RHOC	RND3	ROCK1	SH3PXD2A	SRC	STAT3
G	SVIL	TGFB1	TIMP2	TLN1	VASP	VCL	VEGFA	VIM	WASF1	WASF2	WASL	WIPF1
H	ACTB	B2M	GAPDH	HPRT1	RPLP0	HGDC	NTC	NTC	NTC	PPC	PPC	PPC

Position	Unigene	Refseq	Symbol	Description	Gname
A01	Hs.235750	NM_001102	ACTN1	Actinin, alpha 1	BDPLT15
A02	Hs.737862	NM_001104	ACTN3	Actinin, alpha 3	-
A03	Hs.270291	NM_004924	ACTN4	Actinin, alpha 4	ACTININ-4/FSGS/FSGS1
A04	Hs.643727	NM_005722	ACTR2	ARP2 actin-related protein 2 homolog (yeast)	ARP2
A05	Hs.433512	NM_005721	ACTR3	ARP3 actin-related protein 3 homolog (yeast)	ARP3
A06	Hs.525622	NM_005163	AKT1	V-akt murine thymoma viral oncogene homolog 1	AKT/CWS6/PKB/PKB-ALPHA/PRKBA/RAC/RAC-ALPHA
A07	Hs.525330	NM_001663	ARF6	ADP-ribosylation factor 6	-
A08	Hs.599508	NM_004309	ARHGDIA	Rho GDP dissociation inhibitor (GDI) alpha	GDIA1/HEL-S-47e/NPHS8/RHOGDI/RHOGDI-1
A09	Hs.508738	NM_003899	ARHGEF7	Rho guanine nucleotide exchange factor (GEF) 7	BETA-PIX/COOL-1/COOL1/Nbla10314/P50/P50BP/P85/P85COOL1/P85SPR/PAK3/PIXB
A10	Hs.128316	NM_006340	BAIAP2	BAI1-associated protein 2	BAP2/FLAF3/IRSP53
A11	Hs.479747	NM_014567	BCAR1	Breast cancer anti-estrogen resistance 1	CAS/CAS1/CASS1/CRKAS/P130Cas
A12	Hs.502842	NM_005186	CAPN1	Calpain 1, (mu/I) large subunit	CANP/CANP1/CANPL1/muCANP/muCL
B01	Hs.350899	NM_001748	CAPN2	Calpain 2, (m/II) large subunit	CANP2/CANPL2/CANPml/mCANP
B02	Hs.74034	NM_001753	CAV1	Caveolin 1, caveolae protein, 22kDa	BSCL3/CGL3/LCCNS/MSTP085/PPH3/VIP21
B03	Hs.467637	NM_001791	CDC42	Cell division cycle 42 (GTP binding protein, 25kDa)	CDC42Hs/G25K
B04	Hs.170622	NM_005507	CFL1	Cofilin 1 (non-muscle)	CFL/HEL-S-15/cofilin
B05	Hs.461896	NM_016823	CRK	V-crk sarcoma virus CT10 oncogene homolog (avian)	CRKII/p38
B06	Hs.173894	NM_000757	CSF1	Colony stimulating factor 1 (macrophage)	CSF-1/MCSF
B07	Hs.596164	NM_005231	CTTN	Cortactin	EMS1
B08	Hs.529451	NM_005219	DIAPH1	Diaphanous homolog 1 (Drosophila)	DFNA1/DIA1/DRF1/LFHL1/hDIA1
B09	Hs.368912	NM_001935	DPP4	Dipeptidyl-peptidase 4	ADABP/ADCP2/CD26/DPPIV/TP103
B10	Hs.419815	NM_001963	EGF	Epidermal growth factor	HOMG4/URG
B11	Hs.488293	NM_005228	EGFR	Epidermal growth factor receptor	ERBB/ERBB1/HER1/NISBD2/PIG61/mENA
B12	Hs.497893	NM_001008493	ENAH	Enabled homolog (Drosophila)	ENA/MENA/NDPP1
C01	Hs.487027	NM_003379	EZR	Ezrin	CVIL/CVL/HEL-S-105/VIL2
C02	Hs.654370	NM_004460	FAP	Fibroblast activation protein, alpha	DPPIV/FAPA/SIMP
C03	Hs.284244	NM_002006	FGF2	Fibroblast growth factor 2 (basic)	BFGF/FGF-2/FGFB/HBGF-2
C04	Hs.396530	NM_000601	HGF	Hepatocyte growth factor (hepapoietin A; scatter factor)	DFNB39/F-TCF/HGFB/HPTA/SF
C05	Hs.160562	NM_000618	IGF1	Insulin-like growth factor 1 (somatomedin C)	IGF-I/IGFI/MGF
C06	Hs.643120	NM_000875	IGF1R	Insulin-like growth factor 1 receptor	CD221/IGFIR/IGFR/JTK13
C07	Hs.706355	NM_004517	ILK	Integrin-linked kinase	HEL-S-28/ILK-1/ILK-2/P59/p59ILK
C08	Hs.440955	NM_000885	ITGA4	Integrin, alpha 4 (antigen CD49D, alpha 4 subunit of VLA-4 receptor)	CD49D/IA4
C09	Hs.643813	NM_002211	ITGB1	Integrin, beta 1 (fibronectin receptor, beta polypeptide, antigen CD29 includes MDF2, MSK12)	CD29/FNRB/GPIIA/MDF2/MSK12/VLA-BETA/VLAB
C10	Hs.375957	NM_000211	ITGB2	Integrin, beta 2 (complement component 3 receptor 3 and 4 subunit)	CD18/LAD/LCAMB/LFA-1/MAC-1/MF17/MFI7
C11	Hs.218040	NM_000212	ITGB3	Integrin, beta 3 (platelet glycoprotein IIIa, antigen CD61)	BDPLT16/BDPLT2/CD61/GP3A/GPIIIa/GT
C12	Hs.647035	NM_002314	LIMK1	LIM domain kinase 1	LIMK/LIMK-1
D01	Hs.431850	NM_002745	MAPK1	Mitogen-activated protein kinase 1	ERK/ERK-2/ERK2/ERT1/MAPK2/P42MAPK/PRKM1/PRKM2/p38/p40/p41/p41mapk/p42-MAPK
D02	Hs.132966	NM_000245	MET	Met proto-oncogene (hepatocyte growth factor receptor)	AUTS9/DFNB97/HGFR/RCCP2/c-Met
D03	Hs.2399	NM_004995	MMP14	Matrix metallopeptidase 14 (membrane-inserted)	MMP-14/MMP-X1/MT-MMP/MT-MMP 1/MT1-MMP/MT1MMP/MTMMP1/WNCHRS
D04	Hs.513617	NM_004530	MMP2	Matrix metallopeptidase 2 (gelatinase A, 72kDa gelatinase, 72kDa type IV collagenase)	CLG4/CLG4A/MMP-2/MMP-II/MONA/TBE-1
D05	Hs.297413	NM_004994	MMP9	Matrix metallopeptidase 9 (gelatinase B, 92kDa gelatinase, 92kDa type IV collagenase)	CLG4B/GELB/MANDP2/MMP-9
D06	Hs.713679	NM_002444	MSN	Moesin	HEL70

Position	Unigene	Refseq	Symbol	Description	Gname
D07	Hs.16355	NM_005964	MYH10	Myosin, heavy chain 10, non-muscle	NMMHC-IIB/NMMHCB
D08	Hs.474751	NM_002473	MYH9	Myosin, heavy chain 9, non-muscle	BDPLT6/DFNA17/EPSTS/FTNS/MHA/NMHC-II-A/NMMHC-IIA/NMMHCA
D09	Hs.504687	NM_006097	MYL9	Myosin, light chain 9, regulatory	LC20/MLC-2C/MLC2/MRLC1/MYRL2
D10	Hs.477375	NM_053025	MYLK	Myosin light chain kinase	AAT7/KRP/MLCK/MLCK1/MLCK108/MLCK210/MSTP083/MYLK1/smMLCK
D11	Hs.435714	NM_002576	PAK1	P21 protein (Cdc42/Rac)-activated kinase 1	PAKalpha
D12	Hs.20447	NM_005884	PAK4	P21 protein (Cdc42/Rac)-activated kinase 4	-
E01	Hs.494691	NM_005022	PFN1	Profilin 1	ALS18
E02	Hs.553498	NM_006218	PIK3CA	Phosphoinositide-3-kinase, catalytic, alpha polypeptide	CLOVE/CWS5/MCAP/MCM/MCMTC/PI3K/p110-alpha
E03	Hs.466871	NM_002659	PLAUR	Plasminogen activator, urokinase receptor	CD87/U-PAR/UPAR/URKR
E04	Hs.268177	NM_002660	PLCG1	Phospholipase C, gamma 1	NCKAP3/PLC-II/PLC1/PLC148/PLCgamma1
E05	Hs.732969	NM_002662	PLD1	Phospholipase D1, phosphatidylcholine-specific	-
E06	Hs.531704	NM_002737	PRKCA	Protein kinase C, alpha	AAG6/PKC-alpha/PKCA/PRKACA
E07	Hs.729457	NM_000314	PTEN	Phosphatase and tensin homolog	10q23del/BZS/CWS1/DEC/GLM2/MHAM/MMAC1/PTEN1/TEP1
E08	Hs.395482	NM_005607	PTK2	PTK2 protein tyrosine kinase 2	FADK/FAK/FAK1/FRNK/PPP1R71/p125FAK/pp125FAK
E09	Hs.491322	NM_004103	PTK2B	PTK2B protein tyrosine kinase 2 beta	CADTK/CAKB/FADK2/FAK2/PKB/PTK/PYK2/RAFTK
E10	Hs.417549	NM_002827	PTPN1	Protein tyrosine phosphatase, non-receptor type 1	PTP1B
E11	Hs.446336	NM_002859	PXN	Paxillin	-
E12	Hs.413812	NM_006908	RAC1	Ras-related C3 botulinum toxin substrate 1 (rho family, small GTP binding protein Rac1)	MIG5/Rac-1/TC-25/p21-Rac1
F01	Hs.517601	NM_002872	RAC2	Ras-related C3 botulinum toxin substrate 2 (rho family, small GTP binding protein Rac2)	EN-7/Gx/HSPC022/p21-Rac2
F02	Hs.664080	NM_002890	RASA1	RAS p21 protein activator (GTPase activating protein) 1	CM-AVM/CMAVM/GAP/PKWS/RASA/RASGAP/p120GAP/p120RASGAP
F03	Hs.263671	NM_002906	RDX	Radixin	DFNB24
F04	Hs.247565	NM_000539	RHO	Rhodopsin	CSNBAD1/OPN2/RP4
F05	Hs.247077	NM_001664	RHOA	Ras homolog gene family, member A	ARH12/ARHA/RHO12/RHOH12
F06	Hs.502876	NM_004040	RHOB	Ras homolog gene family, member B	ARH6/ARHB/MST081/MSTP081/RHOH6
F07	Hs.502659	NM_175744	RHOC	Ras homolog gene family, member C	ARH9/ARHC/H9/RHOH9
F08	Hs.6838	NM_005168	RND3	Rho family GTPase 3	ARHE/Rho8/RhoE/memB
F09	Hs.306307	NM_005406	ROCK1	Rho-associated, coiled-coil containing protein kinase 1	P160ROCK/ROCK-I
F10	Hs.678727	NM_014631	SH3PXD2A	SH3 and PX domains 2A	FISH/SH3MD1/TKS5
F11	Hs.195659	NM_005417	SRC	V-src sarcoma (Schmidt-Ruppin A-2) viral oncogene homolog (avian)	ASV/SRC1/c-SRC/p60-Src
F12	Hs.463059	NM_003150	STAT3	Signal transducer and activator of transcription 3 (acute-phase response factor)	ADMIO/APRF/HIES
G01	Hs.499209	NM_003174	SVIL	Supervillin	-
G02	Hs.645227	NM_000660	TGFB1	Transforming growth factor, beta 1	CED/DPD1/LAP/TGFB/TGFbeta
G03	Hs.633514	NM_003255	TIMP2	TIMP metallopeptidase inhibitor 2	CSC-21K/DDC8
G04	Hs.471014	NM_006289	TLN1	Talin 1	ILWEQ/TLN
G05	Hs.515469	NM_003370	VASP	Vasodilator-stimulated phosphoprotein	-
G06	Hs.643896	NM_003373	VCL	Vinculin	CMD1W/CMH15/HEL114/MV/MVCL
G07	Hs.73793	NM_003376	VEGFA	Vascular endothelial growth factor A	MVCD1/VEGF/VPF
G08	Hs.455493	NM_003380	VIM	Vimentin	CTRCT30/HEL113
G09	Hs.75850	NM_003931	WASF1	WAS protein family, member 1	SCAR1/WAVE/WAVE1
G10	Hs.512079	NM_006990	WASF2	WAS protein family, member 2	IMD2/SCAR2/WASF4/WAVE2/dJ393P12.2
G11	Hs.143728	NM_003941	WASL	Wiskott-Aldrich syndrome-like	N-WASP/NWASP/WASPB
G12	Hs.128067	NM_003387	WIPF1	WAS/WASL interacting protein family, member 1	PRPL-2/WAS2/WASPIP/WIP
H01	Hs.520640	NM_001101	ACTB	Actin, beta	BRWS1/PS1TP5BP1

Position	Unigene	Refseq	Symbol	Description	Gname
H02	Hs.534255	NM_004048	B2M	Beta-2-microglobulin	-
H03	Hs.592355	NM_002046	GAPDH	Glyceraldehyde-3-phosphate dehydrogenase	G3PD/GAPD/HEL-S-162eP
H04	Hs.412707	NM_000194	HPRT1	Hypoxanthine phosphoribosyltransferase 1	HGPRT/HPRT
H05	Hs.546285	NM_001002	RPLP0	Ribosomal protein, large, P0	L10E/LP0/P0/PRLP0/RPP0
H06	N/A		HGDC	Human Genomic DNA Contamination	HIGX1A
H07	N/A		NTC	Positive PCR Control	
H08	N/A		NTC		
H09	N/A		NTC		
H10	N/A		PPC	Positive PCR Control	PPC
H11	N/A		PPC	Positive PCR Control	PPC
H12	N/A		PPC	Positive PCR Control	PPC

Symbol	Well	Test Sample											
		20170730mic	20170811mic	20171120mic	exp4	exp5	exp6	exp7	exp8	exp9	exp10	AVG	SD
ACTN1	A01	24.84	24.61	23.23								24.23	0.87
ACTN3	A02	**36.64**	34.55	33.43								34.33	0.81
ACTN4	A03	22.47	22.76	20.82								22.02	1.05
ACTR2	A04	26.77	27.3	23.89								25.99	1.84
ACTR3	A05	25.13	24.81	22.54								24.16	1.41
AKT1	A06	27.73	28.57	25.10								27.13	1.81
ARF6	A07	25.63	25.47	22.48								24.53	1.77
ARHGDIA	A08	25.29	26.21	23.15								24.88	1.57
ARHGEF7	A09	30.74	31.14	27.51								29.80	1.99
BAIAP2	A10	33.02	34.25	30.20								32.49	2.08
BCAR1	A11	29.03	30.11	26.63								28.59	1.78
CAPN1	A12	25.78	25.79	24.13								25.23	0.96
CAPN2	B01	26.88	27.87	24.13								26.29	1.94
CAV1	B02	23.31	23.13	21.14								22.53	1.20
CDC42	B03	25.76	26.17	23.60								25.18	1.38
CFL1	B04	21.62	21.32	20.71								21.22	0.46
CRK	B05	27.23	26.84	24.30								26.12	1.59
CSF1	B06	26.13	27.29	21.90								25.11	2.84
CTTN	B07	27.29	27.57	25.05								26.64	1.38
DIAPH1	B08	27.29	28.17	25.28								26.91	1.48
DPP4	B09	**35.16**	33	30.53								32.84	2.24
EGF	B10	29.88	29.9	29.47								29.75	0.24
EGFR	B11	33.27	32.53	29.08								31.63	2.24
ENAH	B12	29.1	29.45	25.27								27.94	2.32
EZR	C01	26.83	26.82	24.17								25.94	1.53
FAP	C02	28.93	28.24	26.09								27.75	1.48
FGF2	C03	27.64	26.53	23.46								25.88	2.17
HGF	C04	32.15	33.46	29.60								31.74	1.96
IGF1	C05	33.81	**35.32**	31.05								33.29	2.03
IGF1R	C06	30.31	31.76	26.81								29.63	2.54
ILK	C07	25.56	25.77	24.01								25.11	0.96
ITGA4	C08	32.98	33.54	31.10								32.54	1.28
ITGB1	C09	33.95	31.69	30.57								32.07	1.72
ITGB2	C10	24.75	24.65	20.29								23.23	2.55
ITGB3	C11	27.77	29.47	28.04								28.43	0.91
LIMK1	C12	29.72	30.28	26.23								28.74	2.19
MAPK1	D01	31.69	32.02	31.03								31.58	0.50
MET	D02	28.25	28.3	25.18								27.24	1.79
MMP14	D03	30.93	30.64	26.52								29.36	2.47
MMP2	D04	27.77	28.69	24.94								27.13	1.95
MMP9	D05	27.58	27.49	23.88								26.32	2.11
MSN	D06	31.75	34.93	34.50								33.73	1.73
MYH10	D07	28.65	29.92	24.09								27.55	3.07
MYH9	D08	30.44	31.13	26.10								29.22	2.73
MYL9	D09	23.75	23.63	22.24								23.21	0.84
MYLK	D10	22.99	22.83	21.63								22.48	0.74
PAK1	D11	34.8	33.88	30.24								32.97	2.41
PAK4	D12	29.52	30.44	27.35								29.10	1.59
PFN1	E01	28.2	29.06	26.13								27.80	1.51
PIK3CA	E02	22.18	22.42	21.29								21.96	0.60
PLAUR	E03	30.16	30.06	26.44								28.89	2.12
PLCG1	E04	25.46	25.61	23.82								24.96	0.99
PLD1	E05	31.24	31.69	28.04								30.32	1.99
PRKCA	E06	31.69	31.18	28.25								30.37	1.86
PTEN	E07	30.11	30.05	28.24								29.47	1.06
PTK2	E08	26.85	26.88	23.98								25.90	1.67
PTK2B	E09	29.33	30.12	25.87								28.44	2.26
PTPN1	E10	29.2	29.82	28.27								29.10	0.78
PXN	E11	30.3	31.32	26.92								29.51	2.30
RAC1	E12	29.9	31.69	26.33								29.31	2.73
RAC2	F01	25.24	25.24	23.56								24.68	0.97
RASA1	F02	31.86	31.92	29.15								30.98	1.58
RDX	F03	29.49	30.03	25.93								28.48	2.23
RHO	F04	26.81	26.18	24.07								25.69	1.44
RHOA	F05	**36.57**	34.84	32.65								34.16	1.31
RHOB	F06	23.13	23.67	21.01								22.60	1.41
RHOC	F07	26.48	27.33	23.96								25.92	1.75
RND3	F08	23.65	24.17	21.99								23.27	1.14
ROCK1	F09	26.19	25.55	22.66								24.80	1.88
SH3PXD2A	F10	28.56	28.7	25.05								27.44	2.07
SRC	F11	30.25	30.84	26.31								29.13	2.46
STAT3	F12	29.96	29.34	28.86								29.39	0.55

Symbol	Well	Test Sample										
		20170730mic	20170811mic	20171120mic	exp4	exp5	exp6	exp7	exp8	exp9	exp10	AVG	SD
SVIL	G01	25.69	26.33	23.70								25.24	1.37
TGFB1	G02	26.17	26.07	24.12								25.45	1.16
TIMP2	G03	26.62	27.11	23.70								25.81	1.84
TLN1	G04	25.46	25.52	23.02								24.67	1.43
VASP	G05	29.46	30.44	23.83								27.91	3.57
VCL	G06	27.65	28.01	25.88								27.18	1.14
VEGFA	G07	29.09	30.62	24.98								28.23	2.92
VIM	G08	26	26.5	22.30								24.93	2.29
WASF1	G09	30.82	31.57	29.83								30.74	0.87
WASF2	G10	28.9	28.92	26.48								28.10	1.40
WASL	G11	28.81	29.92	25.84								28.19	2.11
WIPF1	G12	27.68	28.14	24.67								26.83	1.88
ACTB	H01	19.03	18.94	17.88								18.62	0.64
B2M	H02	21.3	20.89	19.88								20.69	0.73
GAPDH	H03	27.87	27.71	25.90								27.16	1.09
HPRT1	H04	21.19	21.24	18.04								20.16	1.83
RPLP0	H05	18.96	19.27	18.24								18.82	0.53
HGDC	H06	**36.91**	**37.14**	33.01								34.34	1.15
NTC	H07												
NTC	H08												
NTC	H09												
PPC	H10	24.58	24.7	24.75								24.68	0.09
PPC	H11	24.61	24.7	24.65								24.65	0.05
PPC	H12	24.5	24.72	24.77								24.66	0.14

C_t Range	Distribution of C_t Values										AVG	STD
	exp1	exp2	exp3	exp4	exp5	exp6	exp7	exp8	exp9	exp10		
<25	17	18	46								27	16.5
25-30	51	42	36								43	7.5
30-35	21	31	11								21	10.0
Absent Calls	7	5	3								5	2.0
Percent Distribution of C_t Values												
<25	18%	19%	48%								28%	17%
25-30	53%	44%	38%								45%	8%
30-35	22%	32%	11%								22%	10%
Absent Calls	7%	5%	3%								5%	2%

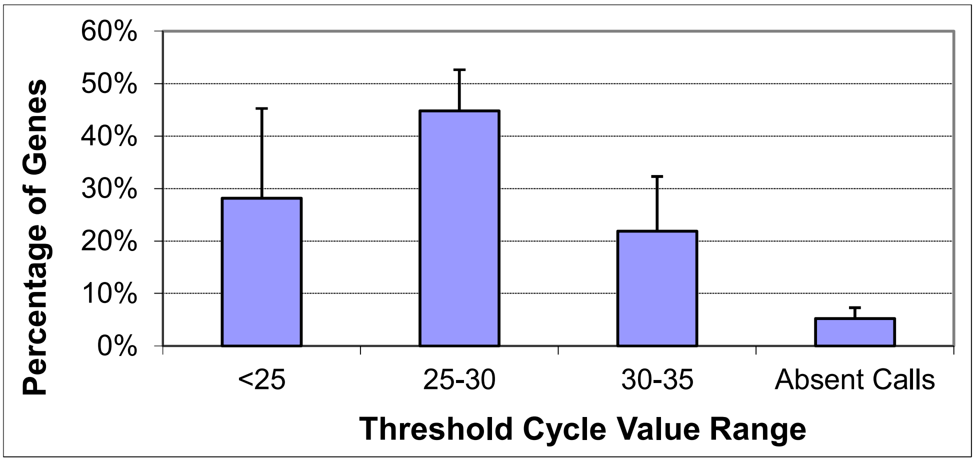

Symbol	Well	Control Sample											
		20170730CK	20170811CK	20171120CK	exp4	exp5	exp6	exp7	exp8	exp9	exp10	AVG	SD
ACTN1	A01	24.62	24.46	22.73								23.94	1.05
ACTN3	A02	35.55	33.08	31.42								33.17	1.79
ACTN4	A03	22.11	21.98	20.48								21.52	0.91
ACTR2	A04	26.44	26.15	23.10								25.23	1.85
ACTR3	A05	24.34	23.54	21.84								23.24	1.28
AKT1	A06	27.04	24.95	24.52								25.50	1.35
ARF6	A07	24.58	23.70	22.13								23.47	1.24
ARHGDIA	A08	25.03	23.71	22.51								23.75	1.26
ARHGEF7	A09	30.33	28.71	27.18								28.74	1.58
BAIAP2	A10	32.57	30.06	28.56								30.40	2.03
BCAR1	A11	29.04	26.79	26.11								27.31	1.53
CAPN1	A12	25.22	25.21	24.04								24.82	0.68
CAPN2	B01	26.64	24.52	23.54								24.90	1.58
CAV1	B02	22.78	21.70	20.56								21.68	1.11
CDC42	B03	24.92	24.62	23.00								24.18	1.03
CFL1	B04	20.56	20.30	20.06								20.31	0.25
CRK	B05	26.01	25.65	24.09								25.25	1.02
CSF1	B06	25.21	22.94	22.67								23.61	1.40
CTTN	B07	26.72	25.32	24.20								25.41	1.26
DIAPH1	B08	26.85	26.70	24.34								25.96	1.41
DPP4	B09	32.61	31.15	29.73								31.16	1.44
EGF	B10	29.68	28.88	28.80								29.12	0.49
EGFR	B11	31.83	29.00	29.45								30.09	1.52
ENAH	B12	28.86	25.72	24.82								26.47	2.12
EZR	C01	25.52	26.04	23.45								25.00	1.37
FAP	C02	27.81	27.01	25.60								26.81	1.12
FGF2	C03	26.17	24.51	23.84								24.84	1.20
HGF	C04	32.12	31.53	28.53								30.73	1.93
IGF1	C05	33.83	34.07	31.48								33.13	1.43
IGF1R	C06	30.32	27.23	25.50								27.68	2.44
ILK	C07	24.89	24.67	23.50								24.35	0.75
ITGA4	C08	33.64	32.90	30.26								32.27	1.78
ITGB1	C09	32.60	30.44	29.26								30.77	1.69
ITGB2	C10	23.97	21.23	20.13								21.78	1.98
ITGB3	C11	28.56	29.45	27.46								28.49	1.00
LIMK1	C12	28.70	29.69	27.66								28.68	1.02
MAPK1	D01	31.53	29.85	30.15								30.51	0.90
MET	D02	27.32	26.34	24.65								26.10	1.35
MMP14	D03	29.76	27.27	25.21								27.41	2.28
MMP2	D04	27.04	26.27	24.63								25.98	1.23
MMP9	D05	26.92	23.76	24.79								25.16	1.61
MSN	D06	31.77	34.81	30.62								32.40	2.16
MYH10	D07	28.09	24.77	22.99								25.28	2.59
MYH9	D08	30.45	27.45	25.98								27.96	2.28
MYL9	D09	23.54	22.52	21.07								22.38	1.24
MYLK	D10	22.72	21.80	20.46								21.66	1.14
PAK1	D11	33.64	29.85	28.84								30.78	2.53
PAK4	D12	29.09	28.31	26.53								27.98	1.31
PFN1	E01	27.85	27.15	25.57								26.86	1.17
PIK3CA	E02	21.50	22.20	20.55								21.42	0.83
PLAUR	E03	29.33	27.62	25.94								27.63	1.70
PLCG1	E04	24.91	26.02	23.92								24.95	1.05
PLD1	E05	30.70	28.64	27.63								28.99	1.56
PRKCA	E06	30.28	29.27	29.31								29.62	0.57
PTEN	E07	29.78	28.57	27.43								28.59	1.18
PTK2	E08	26.45	24.89	24.28								25.21	1.12
PTK2B	E09	29.08	26.82	25.57								27.16	1.78
PTPN1	E10	29.18	29.30	28.26								28.91	0.57
PXN	E11	30.06	28.04	26.13								28.08	1.97
RAC1	E12	29.97	27.04	25.01								27.34	2.49
RAC2	F01	24.50	24.29	22.91								23.90	0.86
RASA1	F02	31.75	30.01	29.50								30.42	1.18
RDX	F03	29.23	27.69	25.62								27.51	1.81
RHO	F04	26.06	25.02	23.72								24.93	1.17
RHOA	F05	34.78	35.90	31.24								33.67	2.11
RHOB	F06	22.81	21.93	20.25								21.66	1.30
RHOC	F07	26.58	24.03	23.27								24.63	1.73
RND3	F08	23.06	22.63	21.73								22.47	0.68
ROCK1	F09	24.89	23.68	23.02								23.86	0.95
SH3PXD2A	F10	28.22	27.14	24.62								26.66	1.85
SRC	F11	30.02	27.01	26.08								27.70	2.06
STAT3	F12	29.52	28.57	28.06								28.72	0.74

Symbol	Well	Control Sample											
		20170730CK	20170811CK	20171120CK	exp4	exp5	exp6	exp7	exp8	exp9	exp10	AVG	SD
SVIL	G01	25.11	24.74	23.58								24.48	0.80
TGFB1	G02	25.43	25.48	23.92								24.94	0.89
TIMP2	G03	26.30	24.34	22.60								24.41	1.85
TLN1	G04	25.08	23.13	22.27								23.49	1.44
VASP	G05	29.04	25.13	22.72								25.63	3.19
VCL	G06	27.50	26.52	25.04								26.35	1.24
VEGFA	G07	29.77	26.03	23.87								26.56	2.99
VIM	G08	25.21	23.72	22.65								23.86	1.29
WASF1	G09	31.22	30.01	29.97								30.40	0.71
WASF2	G10	28.98	28.07	26.34								27.80	1.34
WASL	G11	28.46	26.89	25.21								26.85	1.63
WIPF1	G12	27.00	25.92	24.84								25.92	1.08
ACTB	H01	18.76	18.80	17.08								18.21	0.98
B2M	H02	19.93	20.14	20.17								20.08	0.13
GAPDH	H03	26.74	27.03	25.22								26.33	0.97
HPRT1	H04	20.13	18.79	18.05								18.99	1.05
RPLP0	H05	18.33	18.69	18.26								18.43	0.23
HGDC	H06	**36.12**	**37.33**	31.23								33.74	2.18
NTC	H07												
NTC	H08												
NTC	H09												
PPC	H10	24.75	24.86	24.98								24.86	0.12
PPC	H11	24.75	24.74	24.90								24.80	0.09
PPC	H12	23.73	22.70	24.80								23.74	1.05

C_t Range	Distribution of C_t Values										AVG	STD
	exp1	exp2	exp3	exp4	exp5	exp6	exp7	exp8	exp9	exp10		
**<25**	24	36	52								37	14.0
**25-30**	47	45	34								42	7.0
**30-35**	20	10	7								12	6.8
**Absent Calls**	5	5	3								4	1.2
**Percent Distribution of C_t Values**												
**<25**	25%	38%	54%								39%	15%
**25-30**	49%	47%	35%								44%	7%
**30-35**	21%	10%	7%								13%	7%
**Absent Calls**	5%	5%	3%								5%	1%

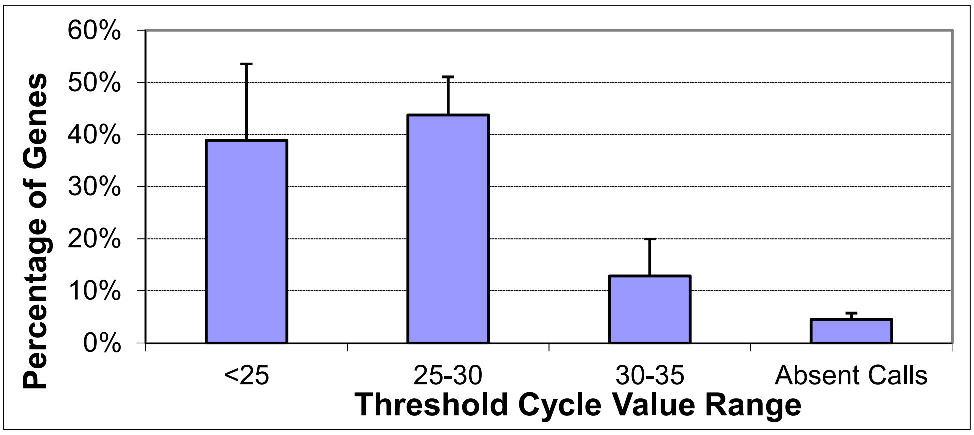

Housekeeping Gene Symbol	Well	Test Sample		
		20170730mic	20170811mic	20171120mic
ACTB	H01	19.03	18.94	17.88
RPLP0	H05	18.96	19.27	18.24
	**AVG**	18.995	19.105	18.06

Housekeeping Gene Symbol	Well	Control Sample		
		20170730CK	20170811CK	20171120CK
ACTB	H01	18.76	18.8	17.08
RPLP0	H05	18.33	18.69	18.26
	**AVG**	18.545	18.745	17.67

Symbol	Well	AVG ΔC_t (Ct(GOI) - Ave Ct		$2^{-\Delta C_t}$		Fold Change	T-TEST	Fold Up- or Down-Regulation	Comments
		Test Sample	Control Sample	Test Sample	Control Sample	Test Sample /Control Sample	p value	Test Sample /Control Sample	
ACTN1	A01	5.51	5.62	2.2E-02	2.0E-02	1.08	0.847027	1.08	OKAY
ACTN3	A02	15.61	14.85	2.0E-05	3.4E-05	**0.59**	0.261094	**-1.69**	B
ACTN4	A03	3.30	3.20	1.0E-01	1.1E-01	0.94	0.850616	-1.07	OKAY
ACTR2	A04	7.27	6.91	6.5E-03	8.3E-03	0.78	0.750695	-1.28	OKAY
ACTR3	A05	5.44	4.92	2.3E-02	3.3E-02	0.70	0.507893	-1.43	OKAY
AKT1	A06	8.41	7.18	2.9E-03	6.9E-03	**0.43**	0.282590	**-2.35**	OKAY
ARF6	A07	5.81	5.15	1.8E-02	2.8E-02	**0.63**	0.616155	**-1.58**	OKAY
ARHGDIA	A08	6.16	5.43	1.4E-02	2.3E-02	**0.60**	0.392588	**-1.66**	OKAY
ARHGEF7	A09	11.08	10.42	4.6E-04	7.3E-04	**0.63**	0.668589	**-1.58**	OKAY
BAIAP2	A10	13.77	12.08	7.2E-05	2.3E-04	**0.31**	0.213536	**-3.23**	B
BCAR1	A11	9.87	8.99	1.1E-03	2.0E-03	**0.54**	0.385396	**-1.84**	OKAY
CAPN1	A12	6.51	6.50	1.1E-02	1.1E-02	0.99	0.939499	-1.01	OKAY
CAPN2	B01	7.57	6.58	5.3E-03	1.0E-02	**0.50**	0.390931	**-1.99**	OKAY
CAV1	B02	3.81	3.36	7.1E-02	9.7E-02	0.73	0.437995	-1.36	OKAY
CDC42	B03	6.46	5.86	1.1E-02	1.7E-02	**0.66**	0.413870	**-1.51**	OKAY
CFL1	B04	2.50	1.99	1.8E-01	2.5E-01	0.70	0.163388	-1.42	OKAY
CRK	B05	7.40	6.93	5.9E-03	8.2E-03	0.72	0.698626	-1.39	OKAY
CSF1	B06	6.39	5.29	1.2E-02	2.6E-02	**0.47**	0.848826	**-2.14**	OKAY
CTTN	B07	7.92	7.09	4.1E-03	7.3E-03	**0.57**	0.278553	**-1.77**	OKAY
DIAPH1	B08	8.19	7.64	3.4E-03	5.0E-03	0.68	0.528554	-1.46	OKAY
DPP4	B09	14.12	12.84	5.6E-05	1.4E-04	**0.41**	0.358651	**-2.43**	B
EGF	B10	11.03	10.80	4.8E-04	5.6E-04	0.85	0.539605	-1.17	OKAY
EGFR	B11	12.91	11.77	1.3E-04	2.9E-04	**0.46**	0.491358	**-2.19**	B
ENAH	B12	9.22	8.15	1.7E-03	3.5E-03	**0.48**	0.459410	**-2.10**	OKAY
EZR	C01	7.22	6.68	6.7E-03	9.7E-03	0.69	0.582549	-1.45	OKAY
FAP	C02	9.03	8.49	1.9E-03	2.8E-03	0.68	0.518679	-1.46	OKAY
FGF2	C03	7.16	6.52	7.0E-03	1.1E-02	**0.64**	0.827430	**-1.55**	OKAY
HGF	C04	13.02	12.41	1.2E-04	1.8E-04	**0.66**	0.622003	**-1.53**	B
IGF1	C05	14.57	14.81	4.1E-05	3.5E-05	1.18	0.638645	1.18	B
IGF1R	C06	10.91	9.36	5.2E-04	1.5E-03	**0.34**	0.328344	**-2.91**	OKAY
ILK	C07	6.39	6.03	1.2E-02	1.5E-02	0.78	0.273902	-1.28	OKAY
ITGA4	C08	13.82	13.95	6.9E-05	6.3E-05	1.09	0.892898	1.09	B
ITGB1	C09	13.35	12.45	9.6E-05	1.8E-04	**0.53**	0.331409	**-1.87**	B
ITGB2	C10	4.51	3.46	4.4E-02	9.1E-02	**0.48**	0.627657	**-2.08**	OKAY
ITGB3	C11	9.71	10.17	1.2E-03	8.7E-04	1.38	0.423004	1.38	OKAY
LIMK1	C12	10.02	10.36	9.6E-04	7.6E-04	1.27	0.516677	1.27	OKAY
MAPK1	D01	12.86	12.19	1.3E-04	2.1E-04	**0.63**	0.323745	**-1.59**	B
MET	D02	8.52	7.78	2.7E-03	4.5E-03	**0.60**	0.546792	**-1.67**	OKAY
MMP14	D03	10.64	9.09	6.3E-04	1.8E-03	**0.34**	0.366564	**-2.93**	OKAY
MMP2	D04	8.41	7.66	2.9E-03	4.9E-03	**0.59**	0.636548	**-1.69**	OKAY
MMP9	D05	7.60	6.84	5.2E-03	8.7E-03	**0.59**	0.584713	**-1.69**	OKAY
MSN	D06	15.01	14.08	3.0E-05	5.8E-05	**0.53**	0.685823	**-1.90**	B
MYH10	D07	8.83	6.96	2.2E-03	8.0E-03	**0.27**	0.383263	**-3.66**	OKAY
MYH9	D08	10.50	9.64	6.9E-04	1.3E-03	**0.55**	0.762574	**-1.82**	OKAY
MYL9	D09	4.49	4.06	4.5E-02	6.0E-02	0.74	0.335211	-1.35	OKAY
MYLK	D10	3.76	3.34	7.4E-02	9.9E-02	0.75	0.299589	-1.34	OKAY
PAK1	D11	14.25	12.46	5.1E-05	1.8E-04	**0.29**	0.229202	**-3.47**	B
PAK4	D12	10.38	9.66	7.5E-04	1.2E-03	**0.60**	0.430920	**-1.65**	OKAY
PFN1	E01	9.08	8.54	1.9E-03	2.7E-03	0.69	0.528321	-1.45	OKAY
PIK3CA	E02	3.24	3.10	1.1E-01	1.2E-01	0.90	0.410644	-1.11	OKAY
PLAUR	E03	10.17	9.31	8.7E-04	1.6E-03	**0.55**	0.592874	**-1.81**	OKAY
PLCG1	E04	6.24	6.63	1.3E-02	1.0E-02	1.31	0.398882	1.31	OKAY
PLD1	E05	11.60	10.67	3.2E-04	6.1E-04	**0.52**	0.468202	**-1.91**	A
PRKCA	E06	11.65	11.30	3.1E-04	4.0E-04	0.78	0.954325	-1.28	A
PTEN	E07	10.75	10.27	5.8E-04	8.1E-04	0.72	0.354003	-1.39	OKAY
PTK2	E08	7.18	6.89	6.9E-03	8.5E-03	0.81	0.843182	-1.23	OKAY
PTK2B	E09	9.72	8.84	1.2E-03	2.2E-03	**0.54**	0.602656	**-1.84**	OKAY
PTPN1	E10	10.38	10.59	7.5E-04	6.5E-04	1.16	0.245040	1.16	OKAY
PXN	E11	10.79	9.76	5.6E-04	1.2E-03	**0.49**	0.518522	**-2.05**	OKAY
RAC1	E12	10.59	9.02	6.5E-04	1.9E-03	**0.34**	0.375235	**-2.96**	OKAY
RAC2	F01	5.96	5.58	1.6E-02	2.1E-02	0.77	0.304314	-1.30	OKAY
RASA1	F02	12.26	12.10	2.0E-04	2.3E-04	0.90	0.900260	-1.11	B
RDX	F03	9.76	9.19	1.2E-03	1.7E-03	0.67	0.812743	-1.48	OKAY
RHO	F04	6.97	6.61	8.0E-03	1.0E-02	0.78	0.668742	-1.28	OKAY
RHOA	F05	15.44	15.35	2.2E-05	2.4E-05	0.94	0.668130	-1.06	B
RHOB	F06	3.88	3.34	6.8E-02	9.9E-02	0.69	0.477047	-1.45	OKAY
RHOC	F07	7.20	6.31	6.8E-03	1.3E-02	**0.54**	0.355561	**-1.86**	OKAY
RND3	F08	4.55	4.15	4.3E-02	5.6E-02	0.76	0.398585	-1.32	OKAY
ROCK1	F09	6.08	5.54	1.5E-02	2.1E-02	0.69	0.800315	-1.45	OKAY
SH3PXD2A	F10	8.72	8.34	2.4E-03	3.1E-03	0.77	0.851284	-1.30	OKAY
SRC	F11	10.41	9.38	7.3E-04	1.5E-03	**0.49**	0.560302	**-2.04**	OKAY

Symbol	Well	AVG ΔC$_t$ (Ct(GOI) - Ave Ct)		2^-ΔC$_t$		Fold Change	T-TEST	Fold Up- or Down-Regulation	Comments
		Test Sample	Control Sample	Test Sample	Control Sample	Test Sample /Control Sample	p value	Test Sample /Control Sample	
STAT3	F12	10.67	10.40	6.2E-04	7.4E-04	0.83	0.497452	-1.21	OKAY
SVIL	G01	6.52	6.16	1.1E-02	1.4E-02	0.78	0.654091	-1.29	OKAY
TGFB1	G02	6.73	6.62	9.4E-03	1.0E-02	0.93	0.907802	-1.08	OKAY
TIMP2	G03	7.09	6.09	7.3E-03	1.5E-02	**0.50**	0.372745	**-2.00**	OKAY
TLN1	G04	5.95	5.17	1.6E-02	2.8E-02	**0.59**	0.325961	**-1.71**	OKAY
VASP	G05	9.19	7.31	1.7E-03	6.3E-03	**0.27**	0.496028	**-3.68**	OKAY
VCL	G06	8.46	8.03	2.8E-03	3.8E-03	0.74	0.430090	-1.34	OKAY
VEGFA	G07	9.51	8.24	1.4E-03	3.3E-03	**0.41**	0.471758	**-2.42**	OKAY
VIM	G08	6.21	5.54	1.3E-02	2.1E-02	**0.63**	0.902025	**-1.59**	OKAY
WASF1	G09	12.02	12.08	2.4E-04	2.3E-04	1.04	0.945265	1.04	B
WASF2	G10	9.38	9.48	1.5E-03	1.4E-03	1.07	0.894387	1.07	OKAY
WASL	G11	9.47	8.53	1.4E-03	2.7E-03	**0.52**	0.521950	**-1.91**	OKAY
WIPF1	G12	8.11	7.60	3.6E-03	5.2E-03	0.70	0.824752	-1.42	OKAY
ACTB	H01	-0.10	-0.11	1.1E+00	1.1E+00	1.00	0.880204	-1.00	OKAY
B2M	H02	1.97	1.76	2.6E-01	3.0E-01	0.86	0.499910	-1.16	OKAY
GAPDH	H03	8.44	8.01	2.9E-03	3.9E-03	0.74	0.373450	-1.35	OKAY
HPRT1	H04	1.44	0.67	3.7E-01	6.3E-01	**0.59**	0.563287	**-1.70**	OKAY
RPLP0	H05	0.10	0.11	9.3E-01	9.3E-01	1.00	0.896841	1.00	OKAY

Legend:

Fold-Change (2^(- Delta Delta Ct)) is the normalized gene expression (2^(- Delta Ct)) in the Test Sample divided the normalized gene expression (2^(- Delta Ct)) in the Control Sample.

Fold-Regulation represents fold-change results in a biologically meaningful way. Fold-change values greater than one indicate a positive- or an up-regulation, and the fold-regulation is equal to the fold-change

Fold-change values less than one indicate a negative or down-regulation, and the fold-regulation is the negative inverse of the fold-change.
Fold-change and fold-regulation values greater than 2 are indicated in red; fold-change values less than 0.5 and fold-regulation values less than -2 are indicated in blue.

p-values: The p values are calculated based on a Student's t-test of the replicate 2^(- Delta Ct) values for each gene in the control group and treatment groups, and p values less than 0.05 are indicated in red.

Comments:

A: This gene's average threshold cycle is relatively high (> 30) in either the control or the test sample, and is reasonably low in the other sample (< 30).

These data mean that the gene's expression is relatively low in one sample and reasonably detected in the other sample suggesting that the actual fold-change value is at least as large as the calculated and reported fold-change result.

This fold-change result may also have greater variations if p value > 0.05; therefore, it is important to have a sufficient number of biological replicates to validate the result for this gene.

B: This gene's average threshold cycle is relatively high (> 30), meaning that its relative expression level is low, in both control and test samples, and the p-value for the fold-change is either unavailable or relatively high (p > 0.05).

This fold-change result may also have greater variations; therefore, it is important to have a sufficient number of biological replicates to validate the result for this gene.

C: This gene's average threshold cycle is either not determined or greater than the defined cut-off (deault 35), in both samples meaning that its expression was undetected, making this fold-change result erroneous and un-interpretable.

Threshold for Fold Difference	2	Threshold for p Value of t-test	0.05

The black line indicates a fold-change in gene expression of 1. The pink lines indicate the desired fold-change in gene expression threshold, as defined by the user in the yellow D1 cell.

The blue line indicates the desired threshold for the p value of the t-test, as defined by user in the yellow I1 cell.

The scale of the X and Y axes can be adjusted by double clicking on them and then re-formating using the standard functions of Microsoft Excel.

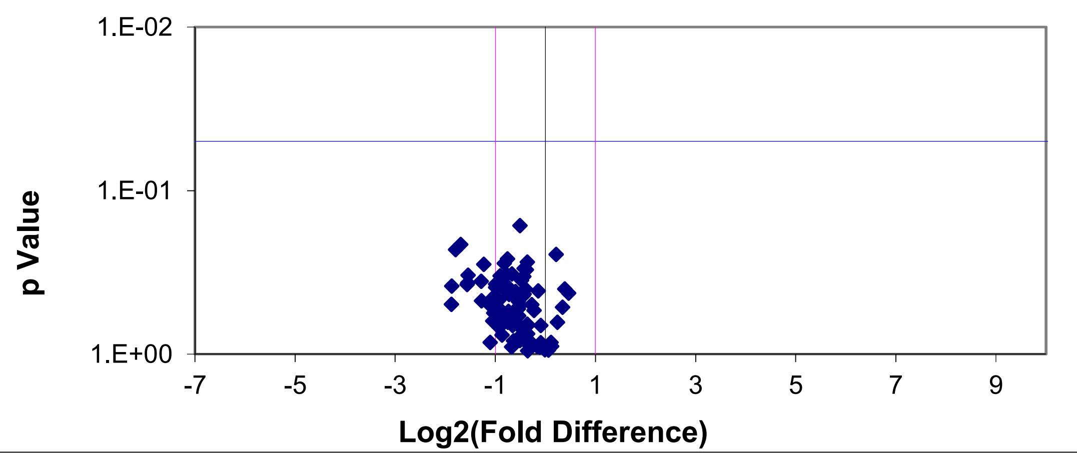

Volcano Plot

Well	Symbol	Log2(FC)	p Value	Comments
A01	ACTN1	0.11	8.5E-01	OKAY
A02	ACTN3	-0.76	2.6E-01	B
A03	ACTN4	-0.09	8.5E-01	OKAY
A04	ACTR2	-0.36	7.5E-01	OKAY
A05	ACTR3	-0.52	5.1E-01	OKAY
A06	AKT1	-1.23	2.8E-01	OKAY
A07	ARF6	-0.66	6.2E-01	OKAY
A08	ARHGDIA	-0.73	3.9E-01	OKAY
A09	ARHGEF7	-0.66	6.7E-01	OKAY
A10	BAIAP2	-1.69	2.1E-01	B
A11	BCAR1	-0.88	3.9E-01	OKAY
A12	CAPN1	-0.01	9.4E-01	OKAY
B01	CAPN2	-0.99	3.9E-01	OKAY
B02	CAV1	-0.45	4.4E-01	OKAY
B03	CDC42	-0.60	4.1E-01	OKAY
B04	CFL1	-0.51	1.6E-01	OKAY
B05	CRK	-0.47	7.0E-01	OKAY
B06	CSF1	-1.10	8.5E-01	OKAY
B07	CTTN	-0.82	2.8E-01	OKAY
B08	DIAPH1	-0.55	5.3E-01	OKAY
B09	DPP4	-1.28	3.6E-01	B
B10	EGF	-0.23	5.4E-01	OKAY
B11	EGFR	-1.13	4.9E-01	B
B12	ENAH	-1.07	4.6E-01	OKAY
C01	EZR	-0.54	5.8E-01	OKAY
C02	FAP	-0.55	5.2E-01	OKAY
C03	FGF2	-0.64	8.3E-01	OKAY
C04	HGF	-0.61	6.2E-01	B
C05	IGF1	0.24	6.4E-01	B
C06	IGF1R	-1.54	3.3E-01	OKAY
C07	ILK	-0.36	2.7E-01	OKAY
C08	ITGA4	0.13	8.9E-01	B
C09	ITGB1	-0.90	3.3E-01	B
C10	ITGB2	-1.05	6.3E-01	OKAY
C11	ITGB3	0.46	4.2E-01	OKAY
C12	LIMK1	0.34	5.2E-01	OKAY
D01	MAPK1	-0.67	3.2E-01	B
D02	MET	-0.74	5.5E-01	OKAY
D03	MMP14	-1.55	3.7E-01	OKAY
D04	MMP2	-0.75	6.4E-01	OKAY
D05	MMP9	-0.76	5.8E-01	OKAY
D06	MSN	-0.93	6.9E-01	B

Well	Symbol	Log2(FC)	p Value	Comments
D07	MYH10	-1.87	3.8E-01	OKAY
D08	MYH9	-0.86	7.6E-01	OKAY
D09	MYL9	-0.43	3.4E-01	OKAY
D10	MYLK	-0.42	3.0E-01	OKAY
D11	PAK1	-1.80	2.3E-01	B
D12	PAK4	-0.73	4.3E-01	OKAY
E01	PFN1	-0.54	5.3E-01	OKAY
E02	PIK3CA	-0.15	4.1E-01	OKAY
E03	PLAUR	-0.86	5.9E-01	OKAY
E04	PLCG1	0.39	4.0E-01	OKAY
E05	PLD1	-0.93	4.7E-01	A
E06	PRKCA	-0.35	9.5E-01	A
E07	PTEN	-0.47	3.5E-01	OKAY
E08	PTK2	-0.30	8.4E-01	OKAY
E09	PTK2B	-0.88	6.0E-01	OKAY
E10	PTPN1	0.22	2.5E-01	OKAY
E11	PXN	-1.04	5.2E-01	OKAY
E12	RAC1	-1.57	3.8E-01	OKAY
F01	RAC2	-0.38	3.0E-01	OKAY
F02	RASA1	-0.16	9.0E-01	B
F03	RDX	-0.57	8.1E-01	OKAY
F04	RHO	-0.35	6.7E-01	OKAY
F05	RHOA	-0.09	6.7E-01	B
F06	RHOB	-0.54	4.8E-01	OKAY
F07	RHOC	-0.90	3.6E-01	OKAY
F08	RND3	-0.40	4.0E-01	OKAY
F09	ROCK1	-0.54	8.0E-01	OKAY
F10	SH3PXD2A	-0.38	8.5E-01	OKAY
F11	SRC	-1.03	5.6E-01	OKAY
F12	STAT3	-0.27	5.0E-01	OKAY
G01	SVIL	-0.36	6.5E-01	OKAY
G02	TGFB1	-0.11	9.1E-01	OKAY
G03	TIMP2	-1.00	3.7E-01	OKAY
G04	TLN1	-0.77	3.3E-01	OKAY
G05	VASP	-1.88	5.0E-01	OKAY
G06	VCL	-0.43	4.3E-01	OKAY
G07	VEGFA	-1.27	4.7E-01	OKAY
G08	VIM	-0.67	9.0E-01	OKAY
G09	WASF1	0.06	9.5E-01	B
G10	WASF2	0.10	8.9E-01	OKAY
G11	WASL	-0.94	5.2E-01	OKAY
G12	WIPF1	-0.51	8.2E-01	OKAY
