# Supplementary material for: Decreased Tiam1‐mediated Rac1 activation is responsible for impaired directional persistence of chondrocyte migration in microtia
Source: J Cell Mol Med. 2024 Jun 4;28(11):e18443. doi: 10.1111/jcmm.18443 (PMC11149491; doi:10.1111/jcmm.18443)
Supplement: Supplementary file 8 — Data S1 [file JCMM-28-e18443-s005.docx]

Caption ：

Movie S1 The high-content imaging of normal and microtia chondrocyte migration trajectories

Movie S2 The OrisTM cell migration imaging of normal and microtia chondrocytes. Scale bars, 200 μm.

Movie S3 The spatiotemporal maps of active Rac1 and cell cytoskeletal changes in microtia chondrocytes with Rac1 Activator II treatment. Scale bars, 20 μm.

Movie S4 The high-content imaging of microtia chondrocyte migration trajectories with Rac1 Activator II treatment

Movie S5 The high-content imaging of microtia chondrocyte migration trajectories after Rac1, Rac1-Q61L and Tiam1 overexpression

Table S2 Cell Motility PCR Array and Data Analysis
